# Biodegradation of polystyrene nanoplastics by *Achromobacter xylosoxidans* M9 offers a mealworm gut-derived solution for plastic pollution

**DOI:** 10.1007/s00203-024-03947-z

**Published:** 2024-04-30

**Authors:** Najat El-Kurdi, Sahar El-Shatoury, Khaled ElBaghdady, Sherif Hammad, Mohamed Ghazy

**Affiliations:** 1 Biotechnology Program, Basic and Applied Science Institute, Egypt-Japan University of Science and Technology, New Burj Al-Arab, Alexandria, Egypt; 2 Aquaculture Biotechnology Department, Fish Farming and Technology Institute, Suez Canal University, Ismailia, Egypt; 3 Biochemistry Department, Faculty of Science, Ain Shams University, Cairo, Egypt; 4 Medicinal Chemistry Department, PharmD Program, Egypt-Japan University of Science and Technology, New Burj Al-Arab, Alexandria, Egypt; 5 Microbiology Department, Faculty of Science, Ain Shams University, Cairo, Egypt; 6 Microbiology Department, Faculty of Science, Suez Canal University, Ismailia, Egypt; 7 Pharmaceutical Chemistry Department, Faculty of Pharmacy, Helwan University, Ain Helwan, Egypt

**Keywords:** Biodegradation, *Tenebrio molitor*, Nanopolystyrene, *Achromobacter xylosoxidans* M9, Nanoplastics

## Abstract

Nanoplastics pose significant environmental problems due to their high mobility and increased toxicity. These particles can cause infertility and inflammation in aquatic organisms, disrupt microbial signaling and act as pollutants carrier. Despite extensive studies on their harmful impact on living organisms, the microbial degradation of nanoplastics is still under research. This study investigated the degradation of nanoplastics by isolating bacteria from the gut microbiome of *Tenebrio molitor* larvae fed various plastic diets. Five bacterial strains capable of degrading polystyrene were identified, with *Achromobacter xylosoxidans* M9 showing significant nanoplastic degradation abilities. Within 6 days, this strain reduced nanoplastic particle size by 92.3%, as confirmed by SEM and TEM analyses, and altered the chemical composition of the nanoplastics, indicating a potential for enhanced bioremediation strategies. The strain also caused a 7% weight loss in polystyrene film over 30 days, demonstrating its efficiency in degrading nanoplastics faster than polystyrene film. These findings might enhance plastic bioremediation strategies.

**Graphical abstract:**

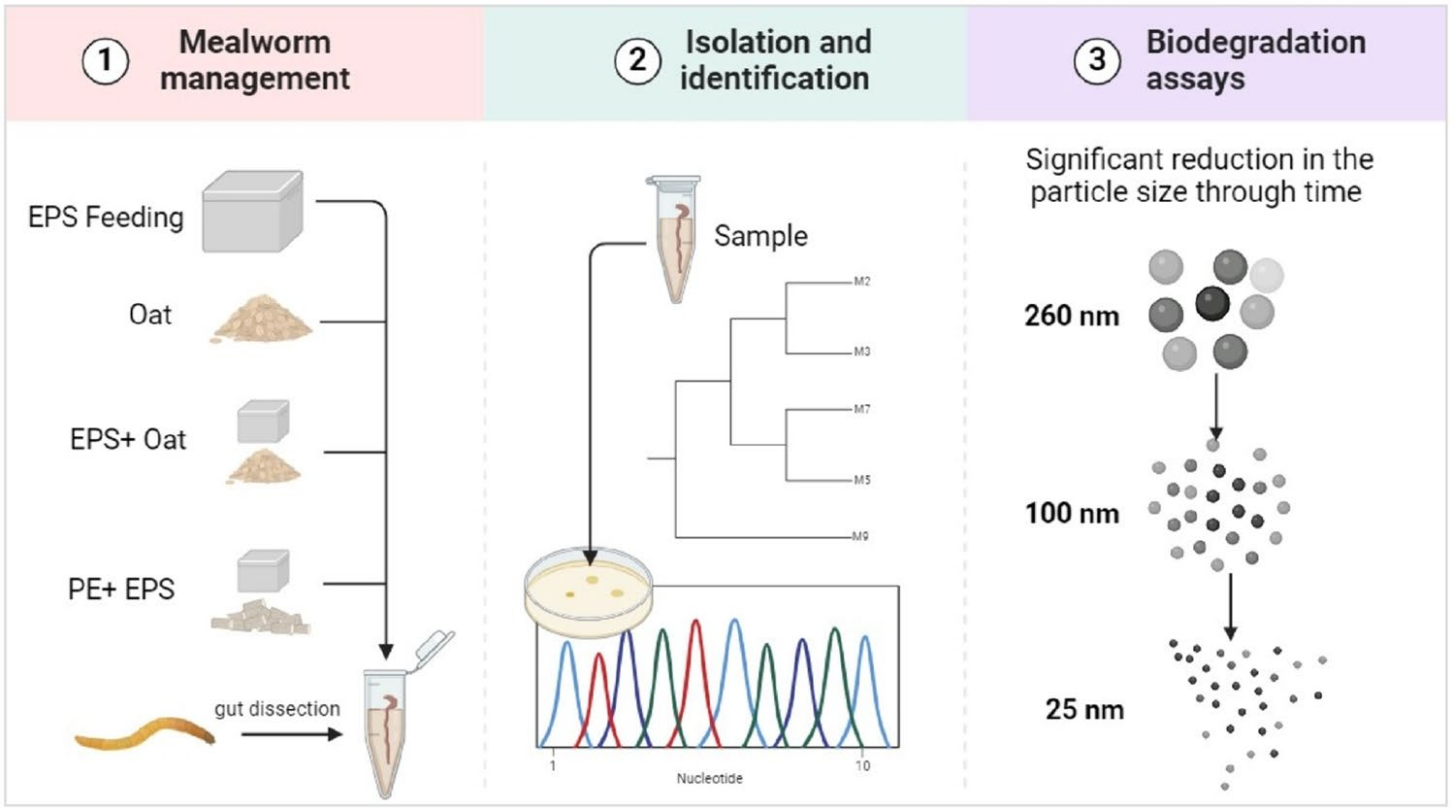

**Supplementary Information:**

The online version contains supplementary material available at 10.1007/s00203-024-03947-z.

## Introduction

Plastics, with their numerous benefits, such as durability, chemical resistance, and lightweight nature, have become ubiquitous in various industries. However, the escalating use of disposable items and the subsequent surge in plastic waste have led to a significant environmental crisis (Mæland and Staupe-Delgado 2020). Polystyrene (PS), a common material in construction, food packaging, and fisheries, has significantly contributed to worldwide microplastic pollution in marine ecosystems (Turner 2020). This pollution stems from the degradation of construction waste, landfill discharge, and other sources, forming nano- and microplastic particles (Kik et al. 2020). These particles, produced through weathering and photodegradation, pose a potential threat to aquatic organisms as they enter the food chain. To combat this, exploring efficient degradation methods to reduce the problem of PS nano- and microplastics is crucial. Therefore, microbial biodegradation emerges as a viable and environmentally friendly solution to the challenges posed by PS nano- and microplastic pollution.

Extensive studies have shed light on the remarkable ability of *Tenebrio molitor* larvae and other invertebrates to consume and degrade plastic materials efficiently. These organisms demonstrate a unique capacity to break down various polymers, effectively metabolizing the resulting substances (Yang et al. 2015a, b, 2020; Urbanek et al. 2020; Yang et al. 2021a; Bulak et al. 2021). Identifying specific bacteria in the gut microbiomes of these invertebrates has proven vital in this process. Prominent strains include *Pseudomonas aeruginosa DSM 50071 *(Kim et al. 2020)*, Klebsiella aerogenes, and Massilia *sp*. FS1903 *(Jiang et al. 2021) play a pivotal role in the decomposition of polystyrene (PS) by facilitating physical and chemical processes. Still, studying the biodegradation of nanoplastics by bacteria is challenging due to their inherent antibacterial properties, which inhibit the growth of many bacteria by causing damage to their cell membranes and production of the reactive oxygen species (ROS) at high nanoplastic concentrations (Shruti et al. 2023). The gut bacteria of the PS-fed mealworm are among the best sources for isolating and identifying nanoplastic decomposers, they adapt to different concentrations of NPS and mineralize them in short periods (Yang et al. 2015b).

Among these bacteria, *Achromobacter xylosoxidans,* Gram-negative bacterium, is known for their adaptability to various aerobic environments such as soil, water, human lungs, and aquatic settings.

While its ability to degrade different xenobiotics such as HDPE (Kowalczyk et al. 2016), LDPE (Maleki Rad et al. 2022), and aromatic hydrocarbons is well documented in the literature (Table S1), its potential for breaking down polystyrene needs more research (Marzec-Grządziel and Gałązka 2023). This bacterium boasts a diverse range of metabolic capabilities, including enhancing plant growth and exhibiting biopesticidal and antifungal properties (Dhaouadi et al. 2019). These findings hint at its potential for beneficial applications beyond pollutant degradation, making it a promising subject for future research.

This preliminary study aims to isolate and identify plastic-degrading bacteria in the gut microbiome of plastic-eating mealworms exposed to EPS and PE.

## Materials and methods

### Chemicals

All mentioned chemicals, solvents and reagents used in this study were of analytical laboratory grade. They were purchased from Sigma Aldrich, ThermoFisher Scientific, Piochem, and Merck.

### Model organism

*Tenebrio molitor* (mealworm) also known as the darkling beetle, Order: *Coleoptera*, family: *Tenebrionidae* Table 1, S2*.* Mealworms, larvae of *T. Molitor* (average weight: 65–75 mg/worm), were obtained from insect breeding farm in Alexandria, Egypt.

**Table 1:** Main features of *Tenebrio molitor* used in this study

	Group 1	Group 2	Group 3	Group 4
Diet	Oats (g)	EPS (g)	Oats + EPS (g)	PE + EPS (g)
Weight	1.8	1.8	0.9 + 0.9	0.9 + 0.9
Larvae initial weight	65 ± 2.3	66 ± 2.5	70 ± 1.3	73 ± 2.7
Larvae number	50			
Color	Yellow			
Shape	a long slender structure and segmented			
Optimum temperature	25 ± 2 °C			
Moisture	70%			
Incubation	In Dark			
Life cycle	Holometabolic insect, includes four life stages: egg, larva, pupa, and imago (or adult)			
Taxonomic classification	Arthropoda, Insecta, Coleoptera, Cucujiformia, Tenebrionidae, Tenebrio (Linnaeus, 1758)			

### Plastic materials

**Fig. 1:**
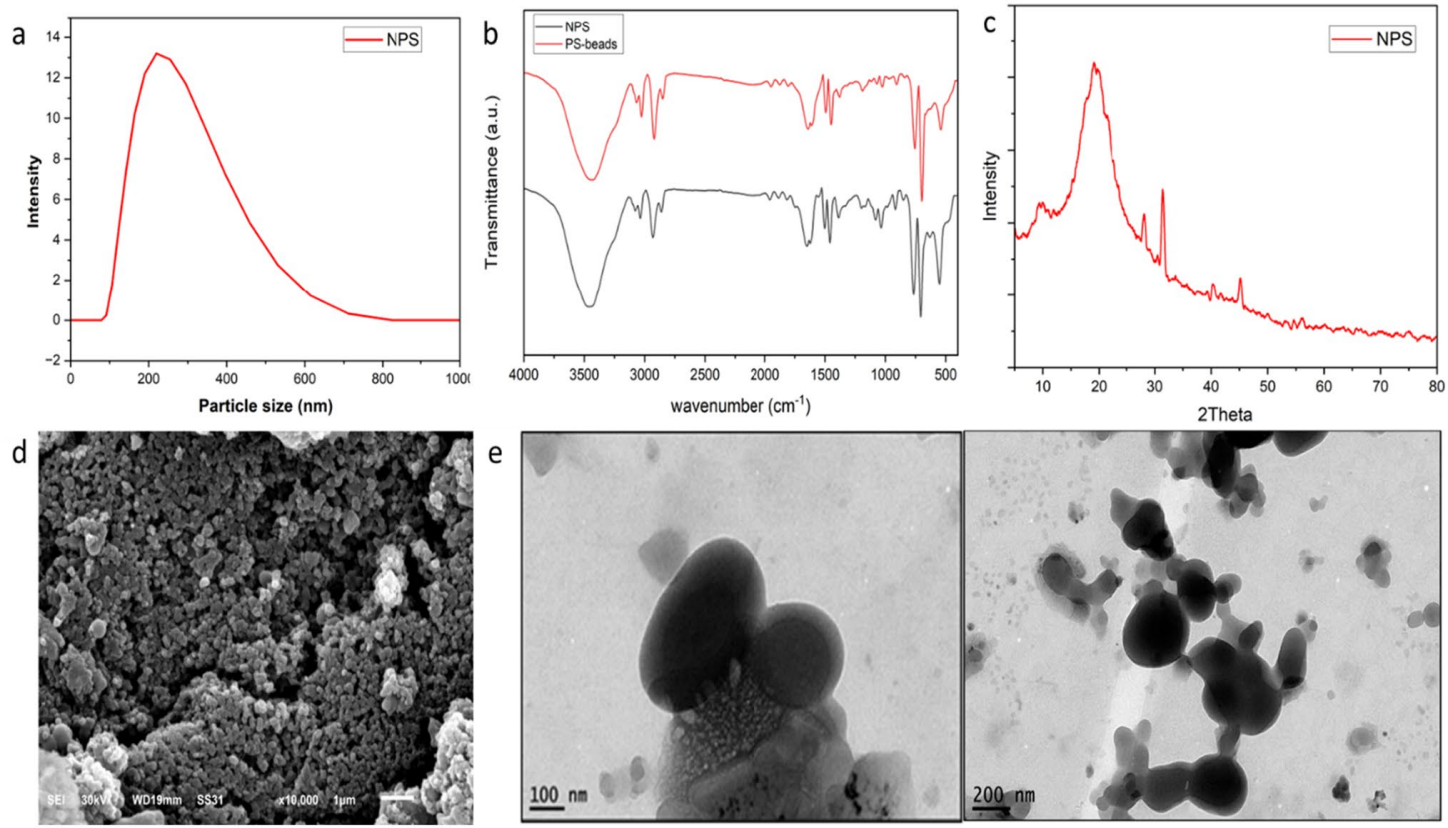
Characterization of NPS **a** Dynamic light scattering analysis **b** ATR-FTIR analysis **c** XRD analysis **d** SEM micrograph e TEM micrograph

For mealworm diet, commercial polyethylene (PE), and Expanded Polystyrene (EPS) Styrofoam were utilized as mealworm food. Purchased from a market in Alexandria, Egypt. Styrofoam was cut into 1 × 1 cm^3^ pieces and cleansed with compressed air to eliminate any remaining residues. For Nano-polystyrene (NPS) synthesis, polystyrene (PS) beads were purchased from (LanXess, China), with densities 0.9, 1.38 and 0.915 g/cm^3^, respectively. The average diameter of plastic beads was 5 mm with colorless regular shape. Nano-polystyrene (NPS) were synthesized using the nanoprecipitation method according to (de Sousa Cunha et al. 2021) Fig S1. The NPS powder were characterized using XRD, DLS, ATR-FTIR, SEM, and TEM techniques to confirm their physical and chemical properties (Fig. 1). Serialization of NPS powder were done by soaking in 70% ethanol (PioChem, Egypt), and allowing them to dry in 60 °C in a clean oven (BioBase, BOV-T140C). Polystyrene film (PSF) was prepared using casting method according to Ebnesajjad (2015). Following its production, the PSF underwent a sterilization process using a conventional technique. The film was initially cut into pieces measuring 5 cm × 5 cm. The previously mentioned films were immersed in a solution of 99.7% anhydrous ethanol for 30 min, guaranteeing complete saturation. Subsequently, they underwent a sequence of three rinses using sterile water to eliminate any remaining ethanol. A sterile filter paper delicately absorbed the films to dehydrate the surface. The PSFs were subsequently placed on a sterile surface within a laminar flow cabinet to ease the process of air drying.

### Mealworm management

**Fig. 2:**
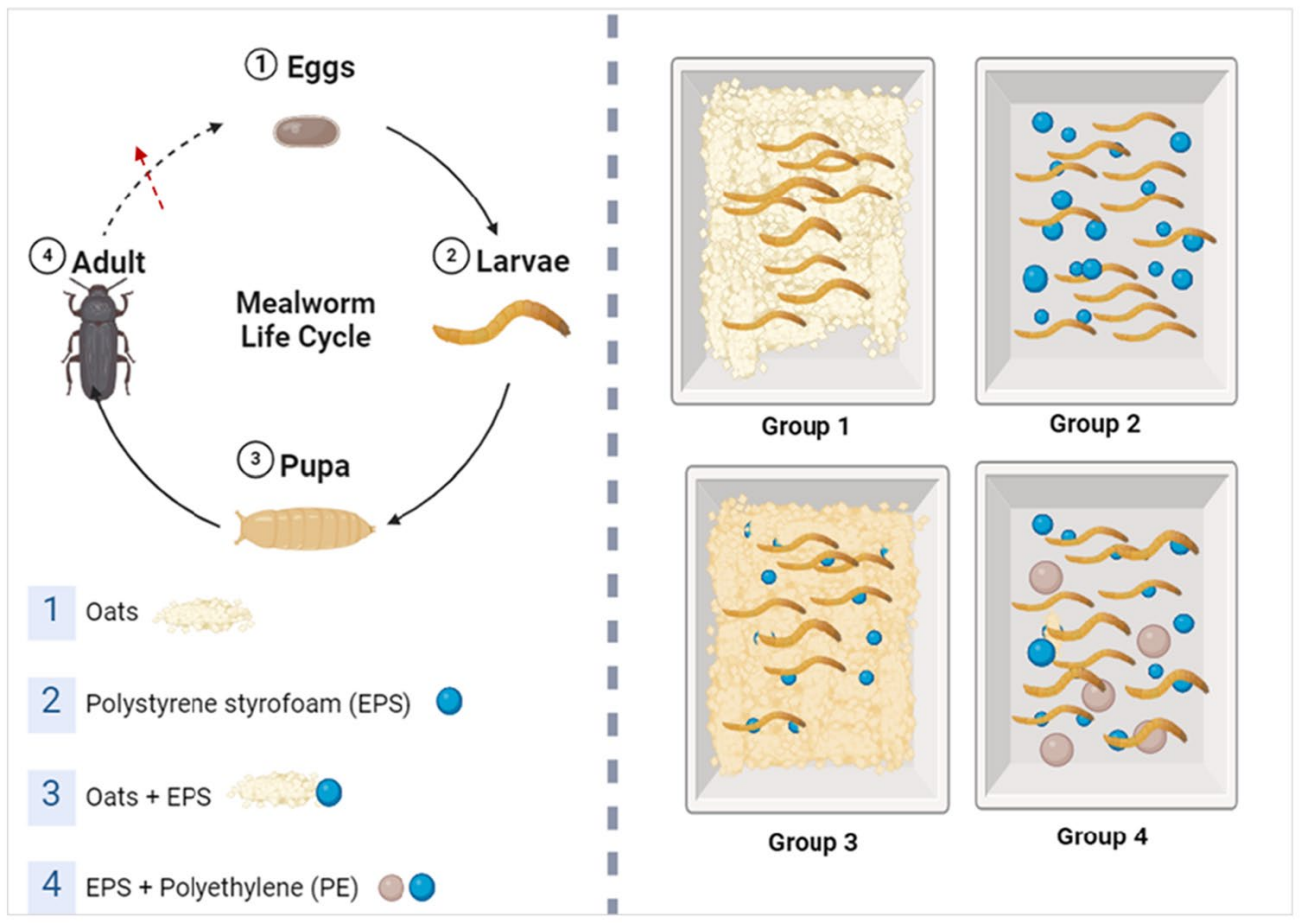
Design of the mealworm experiment (created by www.biorender.com)

Before arrival, the mealworms were given oats; following arrival, they were treated to a 48 h starvation period before starting the experiment with the experimental diet groups. Approximately 50 mealworms were placed in a polypropylene (PP) box with a capacity of 200 mL. The mealworms were then placed in incubators that were kept at a temperature of 25 °C and a humidity level of 70% (Yang et al. 2021b; Bulak et al. 2021; Chen et al. 2023; Cheng et al. 2023). The mealworms were divided into four experimental groups Fig. 2. The control group was fed oats, while the other diet groups were fed expanded polystyrene (EPS) only, EPS + polyethylene (PE), and EPS + Oat. This approach aimed to maximize the possibility of finding various plastic-degrading microbes in the mealworms’ gut and test their impact on the mealworms during a long-term incubation. After the experiment, all mealworms were euthanized and discarded properly.

### Medium for NPS degradation

In this study, NPS (100 mg/L) was the sole carbon source and degradation of it was done in the Carbon-Free Basal Media (CFBM); K_2_HPO_4_ 0.7 g L^−1^, KH_2_PO_4_ 0.7 g L^−1^, MgSO_4_.7H_2_O 0.7 g L^−1^, NH_4_NO_3_ 1.0 g L^−1^, NaCl 0.005 g L^−1^, FeSO_4_.7H_2_O 0.002 g L^−1^, ZnSO_4_.7H_2_O 0.002 g L^−1^, MnSO_4_-H_2_O 0.001 g L^−1^ (Kyaw et al. 2012). Cultures were also grown on Luria–Bertani (LB) broth or agar (NaCl 10 g L^−1^; tryptone 10 g L^−1^; and yeast extract 5 g L^−1^ and; Agar 15 g L^−1^). Media and solutions underwent high-pressure steam sterilization at a temperature of 121 °C, a pressure of 103.4 kPa, and a duration of 15 min using autoclave (DaihanSci, Korea).

### Isolation of NPS degrading bacteria

A sample of 10 larvae was obtained from each experimental group. The intestinal tissue was placed in a 1.5 mL centrifuge tube with 1 mL of normal saline solution and agitated for 5 min using a vortex mixer following sterilization of the larvae. One mL of the homogenized gut suspension was cultured in CFB agar media supplemented with NPS as a sole carbon source using pour plate method. *Escherichia coli* ATCC 25922 was inoculated in the NPS-CFB agar media as negative control. Incubation was done for 6 days at 30 °C, colonies were harvested and subsequently purified on LB agar plates (Yang et al. 2015b).

### Primary screening of isolates

Bacterial isolates grew on NPS-CFB agar media were subjected to primary screening on NPS-CFB broth media. In sterile conditions, 30 mg of NPS powder were added to 300 mL of NPS-CFB broth media. Hundred µL of (10^6^ CFU/mL) of the possible NPS degraders were inoculated equally to the flasks. Positive isolates are expected to emulsify the NPS powder. Uninoculated flasks were used as negative control, emulsified NPS were used as positive control. The experiment was conducted in triplicates.

### Standard plate count

The differentiation between the growth of bacterial cells and the cloudiness caused by Nano-polystyrene (NPS) in the liquid medium poses a challenge. Therefore, the plate count assay is considered the most reliable method for accurately determining the number of viable cells present in a given sample. The bacterial enumeration was performed in accordance with the approach described by Bankier et al. (2018).

### Turbidity assay

Adopting the methodology described by Mohan et al. (2016), we utilized an NPS emulsion with a concentration of (100 mg/L) dissolved in a 20 mM phosphate buffer of pH 6.8 as the experimental substrate. For the assay, 3 mL of this prepared emulsion was combined with 1 mL of culture supernatant. The resultant mixture underwent an initial incubation at 50 °C for a period of 20 min, followed by a subsequent incubation at 30 °C for 4 days. Absorbance measurements were taken at a wavelength of 650 nm both prior to and following the incubation periods. The reduction in turbidity was then quantitatively assessed based on these absorbance readings.

### Secondary screening of isolates

Acid production assay using NPS as the sole source of carbon was used to detect its degradation. The amount of acid produced were quantified according to Eq. (1). Carbon-Free Basal media was supplemented with NPS and Bromocresol purple (BCP) (Roth, Germany) as indicator. The selected bacterial isolates in the primary screening were inoculated, and a yellow color is expected to be formed when the acid is produced. The acidity index was measured as follows. CFB agar media supplemented with 1% sucrose and bromocresol purple (0.12 g/L) BCP was used for comparison. The activity was measured as follows;

1 $${\text{AI}}\;\left( {\text{activity index}} \right) = {\text{Diameter of yellow zone}}\;\left( {{\text{mm}}} \right)--{\text{Colony diameter}}\;\left( {{\text{mm}}} \right)$$

### Biofilm activity

To test the effect on NPS on the production of biofilm by M9 and *E. coli* (negative control) was examined in polystyrene 96-well microtiter plates with 100 μL of LB broth per well. M9 was added to each well until a final density of 10^6^ cells mL^−1^, different concentrations of NPS were applied to M9 and *E. coli* with concentrations (0, 50, 100, 300, 500 mg/L). The plates were then incubated at 30 °C for 24 h. Following the incubation period, the medium was discarded, and the biofilm that had formed was rinsed with distilled water. The crystal violet (CV) staining method according to Mor and Sivan (2008) was utilized to determine the population density of the biofilm on the polystyrene surface. The approach employed in this study relies on the established relationship between bacterial biomass and the absorbance (measured at 600 nm) of the ethanol extract derived from the cells. A 0.2% CV solution in 95% ethanol, and the plates were incubated at room temperature for a duration of 15 min. After disposing of the staining solution, the plates were rinsed with distilled water to eliminate any remaining stain. Subsequently, a solution of 95% ethanol was introduced into the well in order to extract the stain from the bacteria that were attached to the surface of the well.

### Weight loss of the PS film

The PS film, which had been incubated with microorganisms for 30 days at 30 °C, was subsequently recovered and subjected to surface sterilization using ethanol and distilled water. The film was subsequently dried in the air, and the remaining weight was measured (Mor and Sivan 2008). The weight of the residual film was determined using the formula:

$${\text{Percentage weight loss }}\left( \% \right)\; = \;\left( {\left( {{\text{Initial film weight}}---{\text{Final film weight}}} \right)/{\text{Initial film weight}}} \right)) \, * \, 100.$$

### Genomic DNA extraction and identification

The genomic DNA of the plastic degrading strains was extracted manually, according to Maloy (1990). The universal primers 27F 5′-CAGAGTTTGATCCTGGCT 3′ and 1492R5′-GGTTTTTTTTACGACTT-3′ were used for PCR amplification of the 16SrDNA region. The PCR conditions given were 37 cycles of 3 min at 94 °C, 1 min at 55 °C, and 1 min at 72 °C. The amplified products were separated by electrophoresis, purified, and sequenced (Macrogen, Korea). The obtained sequences were purified, assembled, and analyzed against the existing sequences on the NCBI database using the BLAST tool. The strains with similar identities were selected for constructing a phylogenetic tree using MEGA 11 software. The obtained strains were deposited in the NCBI with accession numbers PP126564 (*Pseudomonas aeruginosa* M2); PP126565 (*Pseudomonas stutzeri* M3); PP126566 (*Pseudomonas* M7); PP126567 (*Pseudomonas aeruginosa* M8); OR859752 (*Achromobacter xylosoxidans* M9).

### Screening for enzymatic activity

The promising isolates were screened for their possible enzymatic activity. Various types of extracellular enzymes, including cellulases, esterases, lipases, proteases, and laccases, were evaluated for their activity against bacteria capable of grow on NPS emulsion as a sole carbon source. Each enzyme was tested with a specific substrate: carboxymethyl cellulose (CMC) for cellulases, Tween 20 for esterases, Tween 80 for lipases, casein for proteases, and guaiacol for laccases. The assays were carried out according to the following procedure: Cellulase enzyme activity was screened according to Kim et al. (2005). Concisely, bacterial samples were streaked on the surface of the carboxymethyl cellulose agar media plate (0.5 g KH_2_PO_4_, 0.25 g MgSO_4_, 0.25 g cellulose, 15 g agar, and 2 g gelatin), subsequently, the samples were placed in an incubator and maintained at a temperature of 37 °C for a duration of 24 h. Subsequently, the plates were saturated with iodine solution using a dropper. After a few minutes, the plates were examined to determine the presence of the hydrolysis zone. Esterase and lipase enzymes were screened according to Kumar et al. (2012). In brief, the hydrolytic activity of plastic degrading bacterial lipase and esterase were done on tween 80 and tween 20 medium, respectively composed of (g/L): peptone, 10; NaCl, 5; CaCl_2_.2H_2_O, 0.1; agar–agar, 20; tween 20 or tween 80, 10 mL (v/v); white precipitation is expected to be formed. Protease enzyme was tested according to Vijayaraghavan and Vincent (2013). The plates were solidified for 30 min, and holes (3 mm diameter) were made. A crude culture supernatant from nanoplastic-degrading bacteria extracted from the mealworm gut was loaded into the holes. These plates were incubated overnight at 37 °C. For the laccase enzyme, the positive isolates were detected with the formation of reddish brown and screened on guaiacol agar media supplemented with 0.01%(v/v) guaiacol as laccase substrates and 0.35 mM CuSO_4_. Strains were inoculated and incubated overnight at 37 °C (Mandic et al. 2019). The experiments were conducted in triplicates.

### Extraction of nanoplastics

**Fig. 3:**
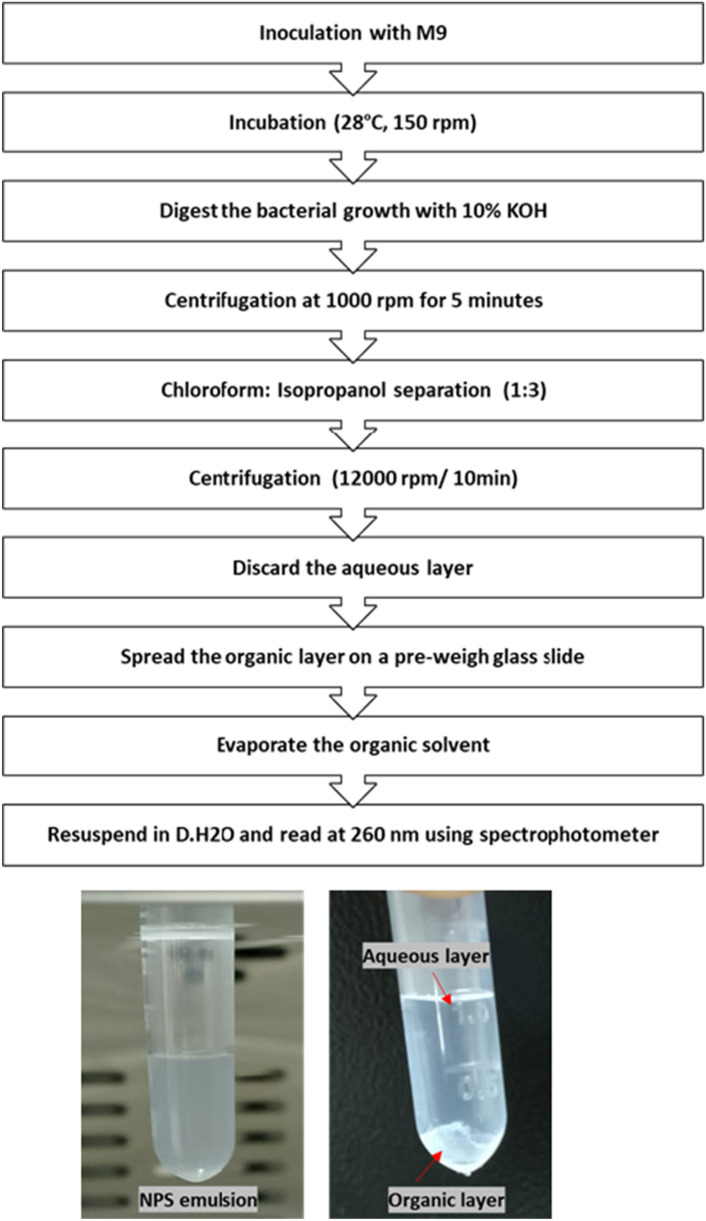
Extraction method of NPS from LCFBM

This extraction process is a physical method used to isolate nanoplastics from complex matrices such as biological cells for further analysis. It doesn’t alter the chemical structure of the nanoplastics but rather utilizes the solubility principles to separate them from other materials. In this method microbial digestion using 10% KOH for 6 h at 40 °C was efficient for digestion of microbial cells and didn’t affect the structure of the nanoplastics (Dehaut et al. 2016). Then, the digestion method was followed by centrifugation at 2000 rpm for 2 min to remove any excess of cell debris in the media. Nanoplastics were extracted according to Manaka et al. (2023). To 10 mL of the sample, add 500 µL of the chloroform mixture (chloroform: isopropanol = 1:3). The mixture was vortexed for 2 min, and then centrifuged at 12,000 rpm for 15 min. The upper aqueous layer was discarded and the solvent layer was allowed to dry to evaporate the solvent. The obtained nanoplastics were subjected to Particle count analysis (PCA), Scanning Electron microscopy (SEM), and ATR-FTIR analysis (Fig. 3).

### Particle count analysis (PCA)

Nanoplastics were examined using an inverted microscope (IM) to determine the particle count and size variations. First, 50 µl of nanoplastic extract was spread to a glass slide and dried. After calibrating the scale, all samples were examined under the inverted microscope. Using an inverted Nikon Eclipse TS100-F microscope coupled with a camera (Ikegami ICD-848P), digital images of all fields of view were captured in transmitted light (all objects appeared as silhouettes). ImageJ software was used to process the obtained high-resolution images to analyze the particle count.

$${\text{Particle count loss }}\left( \% \right)\; = \;\left( {\left( {{\text{Initial particle number}} - {\text{Final particle number}}} \right)/\left( {\text{Initial particle number}} \right)} \right) \, *100.$$

### Particle size analysis

The Nanosizer, by Malvern, United Kingdom, was employed to determine the mean diameter (Dp) and degree of size variation (DPI) of precipitated PS nanoparticles. The measurements were performed at a temperature of 25 °C, with a reading angle of 90° and a wavelength of 633 nm. The obtained PS particles were dispersed in distilled water and then exposed to sonication using a Model 500 Ultrasonic Dismembrator from Fisher Scientific, USA, to break up any present aggregates (de Sousa Cunha et al. 2021).

### Scanning electron microscopy (SEM)

The surface structure and characterization of the NPS were examined before and after treatment using a scanning electron microscope (SEM, Philips-X LP30, FEI company, OR, USA). NPS was obtained using the previously mentioned method. The NPS were conserved using vapor fixation in a tightly sealed container at a temperature of 25 °C for 2 days. The samples were subjected to gold-coating using BAL-TEC-SCDOOS. In addition, the distortions observed on the polystyrene film were analyzed following a 30-day incubation period.

### ATR-Fourier transform infrared analysis (ATR-FTIR)

The infrared absorption spectra were captured using the Satellite 5000 infrared spectrophotometer (FTIR, Thermo, USA). The reference gas used was air, and the spectral scan covered the range of 400–4000 cm^−1^. The vibration spectra were obtained using the attenuated total reflection (ATR) mode of FTIR spectroscopy, with a resolution of 4 cm^−1^, aiming to examine the deterioration and identify the changes in functional groups on the surface of the NPS (Zhang et al. 2023).

### Gas chromatography-mass spectroscopy (GCMS) analysis

The residual medium was extracted with ethyl acetate HPLC grade (Merck, USA) for the detection of the NPS degradation products employing GC–MS (Agilent 6890N, USA). GC conditions were as follows: an injection temperature of 250 °C, an initial column temperature of 80 °C, a hold time of 4 min, increased to 300 °C at the rate of 10 °C per min. The carrier gas He, was injected at a flow rate of 0.7 mL/min and scan range was m/z 40–450.

### Statistical analysis

Analysis of variance (ANOVA) was conducted using SPSS 23.0 (SPSS Inc., Chicago, United States) to evaluate the differences in plastic consumption by mealworms, turbidity assay, biofilm formation and particle count analysis. The experiments were performed in triplicates. Pairwise comparisons were carried out using the student’s t-test and Duncan’s multiple comparison test. The error values are expressed as mean ± standard deviation.

## Results

### Analysis of the feeding behavior of *T. molitor*

**Fig. 4:**
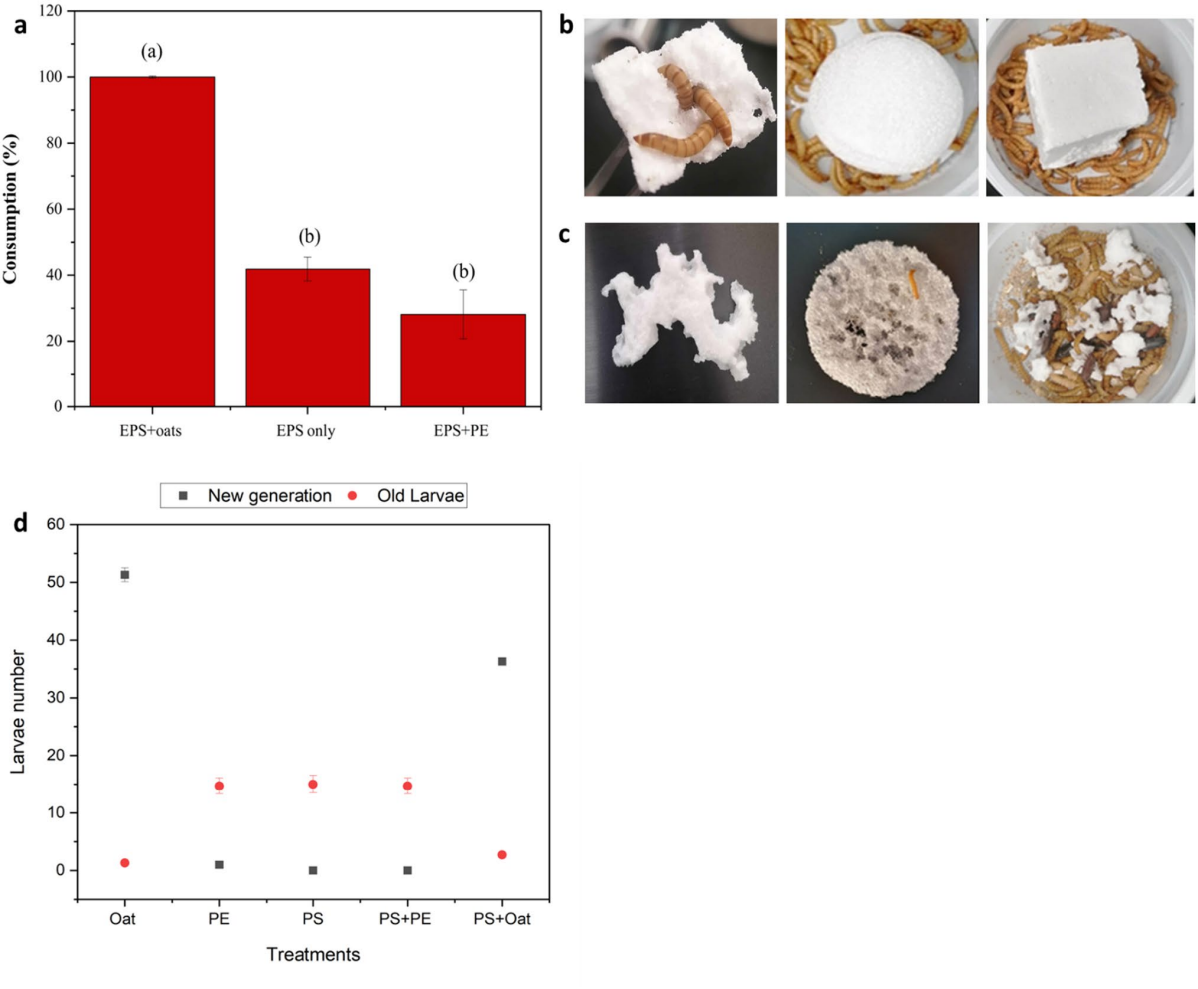
Biodegradation of Expanded Polystyrene (EPS) by *Tenebrio molitor* **a** Consumption of EPS after exposure to ~ 50 *Tenebrio molitor* at the end of the experiment. **b** Styrofoam before the experiment **c** Alterations occurred in the Styrofoam after chewing by *T. molitor.* **d** Number of new larvae generated after 3 months. The figure presents the mean values together with their standard deviations (n = 3). The letters displayed on the bars indicate significant differences among the treatments, as determined by Duncan’s multiple comparison test (*P* < 0.05). (EPS: Expanded polystyrene foam, PE: Polyethylene foam)

Larvae could feed on Expanded Polystyrene Styrofoam (EPS) in each diet group and stay alive throughout 3 months. However, their feeding behavior changed from one group to another. Throughout the experiment, mealworm larvae attacked EPS by chewing and digging in the styrofoam. Holes and discolorations were observed in the 1 days of incubation (Fig. 4b, c). Every day, the containers accumulated frass mixed with micro and nanoplastic powder as a result of the chewing process. Consumption of EPS in each diet group showed a pattern of preference; for example, when EPS was mixed with oats, larvae showed 99.81 ± 0.32% consumption of the EPS. However, when it was the sole source of diet, only 41.9 ± 3.6% were consumed. When it mixed with another polymer their consumption dropped to 28.1 ± 7.40% in this study (Fig. 4a).

Interesting observations were reported; after 3 months of incubation, all larvae in the control group completed their life cycle with 83 ± 4.5% pupation rate and formed eggs then new larvae. On the contrary in the EPS diet groups, the larval reproduction changed from one group to the other. In the diet contained EPS alone, larvae showed an obvious delay in their pupation and transformation to an adult. In the EPS mixed with oats, they completed their life cycle and formed next larval generation, however it was significantly less in number compared to the control group. In the group of two polymers mixture, the larvae matured into adults, but unlike the first two groups, they did not produce offspring, suggesting infertility (Fig. 4c).

The larvae of the EPS alone diet exhibited unusual behaviors compared to those fed on oats, such as accelerated movement, colliding with the container walls, and moving in circles. Additionally, they ceased feeding.

### Isolation of NPS-degrading strains

Nine bacterial strains emerged on the carbon-free basal agar medium (CFBAM) supplemented with NPS, while the control group showed no microbial growth. The presence of bacterial colonies on the NPS plates from yellow mealworms suggesting their ability to degrade polystyrene. Through cultivation and purification efforts, pure colonies were obtained and labeled M1–9. Gram staining revealed these strains as Gram-negative bacteria. On CFBAM, the colony characteristics differed: M2 formed circular, smooth-edged colonies in a light orange hue; M3’s colonies were pale pink with a velvety surface; M7 and M8 developed soft, creamy colonies; while M9 produced dark orange, sticky colonies. All strains showed significant growth and were motile on the agar. Growth on LB media brought out more differences in colony morphology: M2, M3, and M8 presented as transparent, smooth, highly sticky, and mucoidal; M7 was characterized by a rough, wrinkled texture, creamy hue, and solid form; whereas M9 had smooth, circular colonies with a creamy shade and fluffy edges.

**Fig. 5:**
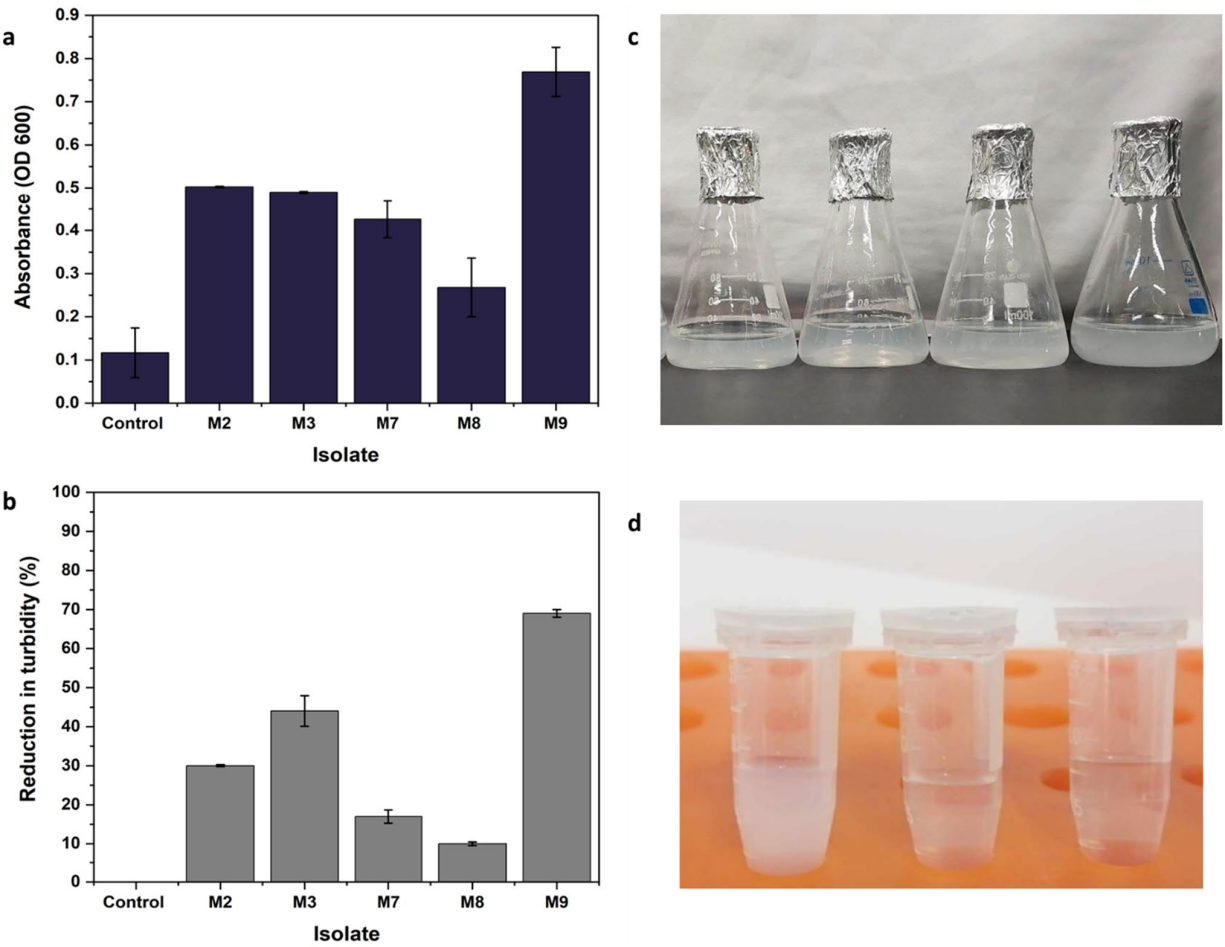
**a**, **c** Growth of bacterial isolates on liquid NPS-basal media **b**, **d** Reduction in the turbidity of the promising NPS degrading bacteria

A growth curve was generated for the most prominent isolates to track changes in optical density at 600 nm (OD600) (Fig. 5a). Bacteria capable of degrading polystyrene caused the NPS powder to emulsify, resulting in turbidity within the media, unlike the control group where the NPS powder remained afloat and the media remained clear. Out of the five bacterial isolates, *A. xylosoxidans M9* exhibited a significantly higher growth rate than the other strains (*P* < 0.05). All isolates-initiated growth within the first 24 h, with distinct variations in growth patterns observed over time. In contrast, the OD_600_ reading of the control group *Escherichia coli* DH5 alpha remained unchanged throughout the experiment. In this study, isolate M9 demonstrated the ability to grow on LCFBM when supplemented with NPS as sole source of carbon, reaching an OD_600_ of 0.8 (Fig. 5a).

### Secondary screening of NPS degrading

Secondary screening of the five bacterial isolates using turbidity assay showed that *Pseudomonas aeruginosa M2*, *Pseudomonas stutzeri M3*, and *Achromobacter xylosoxidans M9* had the best turbidity removal with percentages 30%, 44%, and 56%, respectively (Fig. 5b). The findings revealed that *Achromobacter xylosoxidans M9* had the highest capacity for eliminating NPS in comparison to the other strains. Additionally, *Pseudomonas aeruginosa M2* and *Achromobacter xylosoxidans M9* demonstrated the ability to generate acid in the presence of NPS, however, they were unable to produce acid when grown on a sucrose (Table 2).

**Table 2:** Acid production assay with NPS and sucrose as sole carbon sources

Strain code	NPS ± SD (mm)	Sucrose ± SD (mm)
Control (*E. coli*)	0	1 ± 0.12
M2	10 ± 0.1	0
M3	0	0
M7	30 ± 1	4 ± 0.5
M8	12 ± 1.2	7 ± 0.2
**M9**	**33 ± 2**	**0**

Bold values indicate higher values and the most important components in the table

The qualitative enzymatic assays depicted that isolates *Pseudomonas aeruginosa M2*, and *Achromobacter xylosoxidans M9* were positive for laccases and esterase, *Pseudomonas* sp *M7* was notable for its cellulase and esterase enzymes while *Pseudomonas aeruginosa M8* could produce lipases, esterase and laccase (Table 3).

**Table 3:** Enzymatic activity of the NPS degrading gut bacteria

Strain code	Lipase (Tween 80)	Esterase (Tween 20)	Laccase	Catalase	Oxidase	Protease	Cellulase
M2	++	+++	++	+++	++	+	−
M3	+	+	−	++	++	+	+
M7	−	+	−	+++	++	−	+++
M8	++	+++	++	+++	++	++	−
M9	−	++	+++	+++	+++	−	++

(−) negative activity (+) partially positive (< 2 mm), (++) strong (5–15 mm), (+++) very strong (20–25 mm)

### Growth behaviors of *A. xylosoxidans* M9

**Fig. 6:**
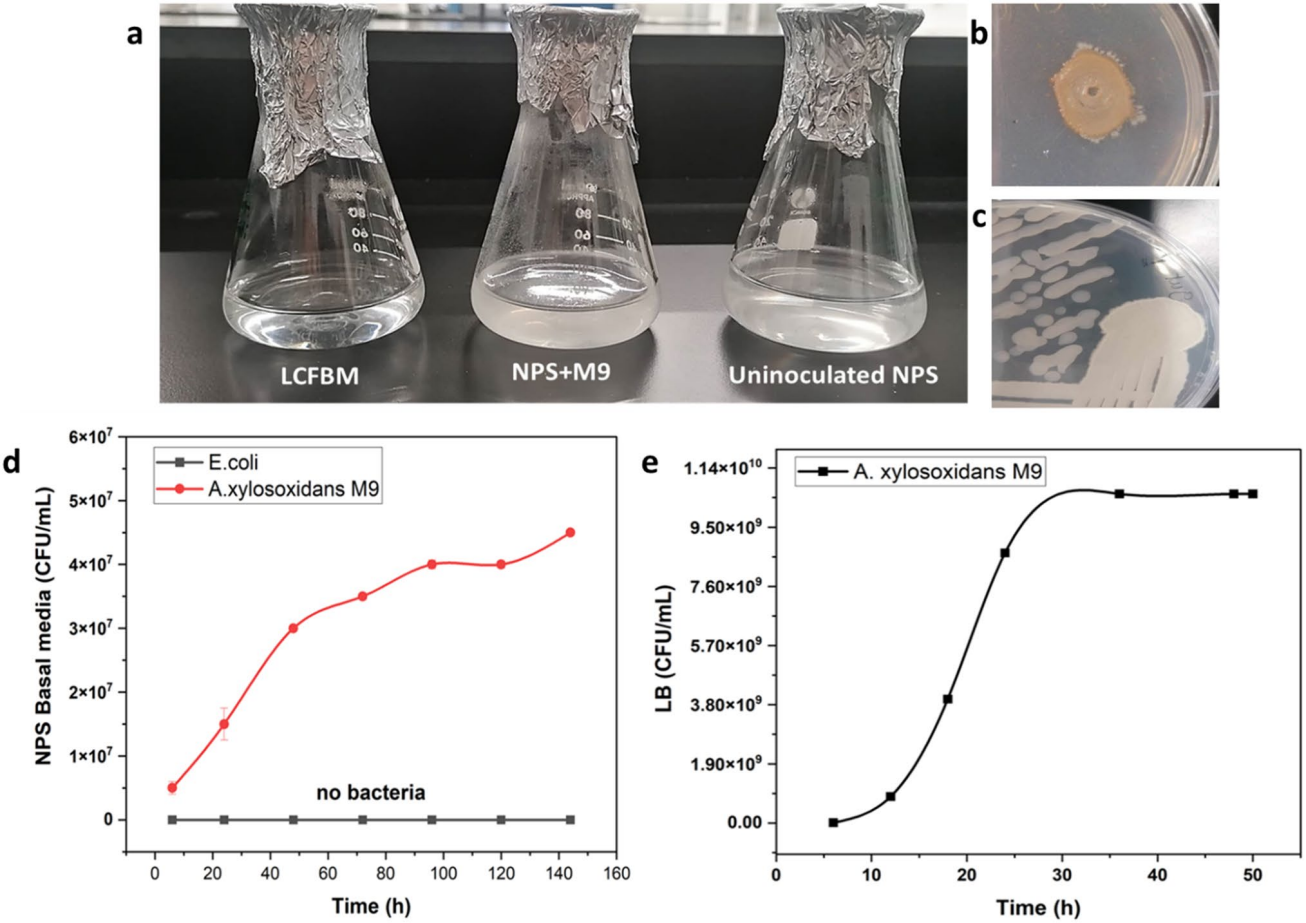
**a** Growth of *A. xylosoxidans* M9 on NPS basal liquid media; **b** M9 on NPS basal agar media; **c** M9 on LB agar; **d** growth curve of M9 on NPS basal media; **e** growth curve of M9 on LB media

The growth rate of *Achromobacter xylosoxidans M9* cultured on carbon-free media supplemented with NPS is approximately 0.063 per hour. This value indicates the rate at which the bacterial population increases in number during the exponential phase of growth under these conditions. On the other hand, the growth rate of the *Achromobacter xylosoxidans M9* cultured on LB media is 1.258 per hour. This indicates a rapid increase in the bacterial population during the 6-h period, reflecting a highly efficient use of available nutrients or an optimal growth condition for the bacteria (Fig. 6).

### Weight loss of the PS film

Among the strains tested, M9 exhibited the highest efficacy, achieving a 7% reduction in PS film weight, thereby indicating its superior capability in degrading polystyrene. In contrast, strains M3 and M8 showed minimal degradation abilities, with weight loss percentages of 1.5% and 1.2%, respectively. The control group, with no weight loss, served as a baseline, confirming that the observed reductions were due to microbial activity (Table 4).

**Table 4:** Weight loss (%) of the PS film

Strain code	Initial weight (g)	Final weight (g) ± SD	Percentage (%) of reduction
Control	0.7	0.7	0
M2	0.7	0.69 ± 0.08	6
M3	0.7	0.641 ± 0.015	1.5
M7	0.7	0.65 ± 0.03	5
M8	0.7	0.688 ± 0.01	1.2
**M9**	**0.7**	**0.63 ± 0.02**	**7**

Bold values indicate higher values and the most important components in the table

### Scanning electron microscopy of PS film

**Fig. 7:**
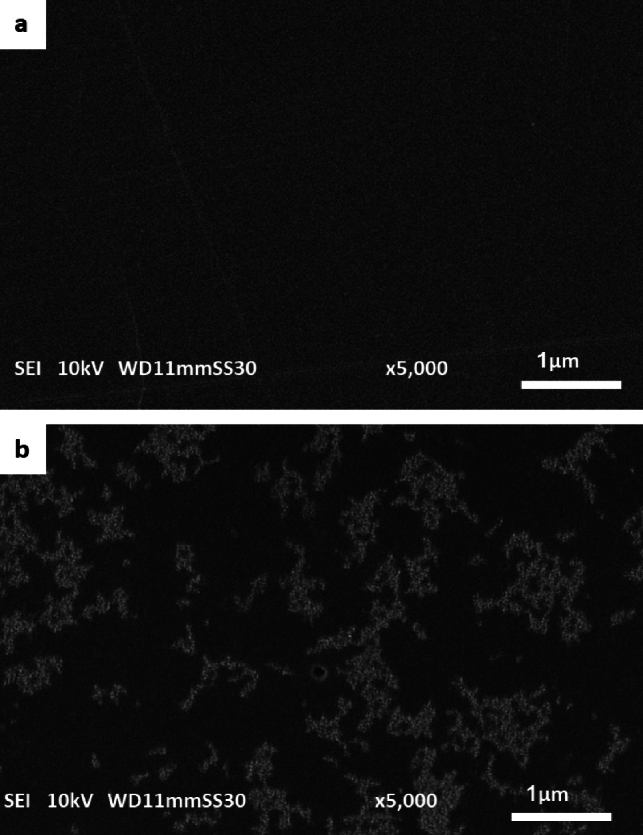
Scanning electron microscope micrograph of the PSF **a** before; and **b** after growth of M9 (scale 1 µm and × 5000 magnification)

The establishment of microbial biofilms on the PS film surfaces further supported the biodegradation process, as evidenced by SEM analyses, which showed significant surface alterations including the formation of cracks, holes, and increased pore size after 30 days of exposure to strain M9 (Fig. 7).

### Identification of NPS-degrading strains

**Fig. 8:**
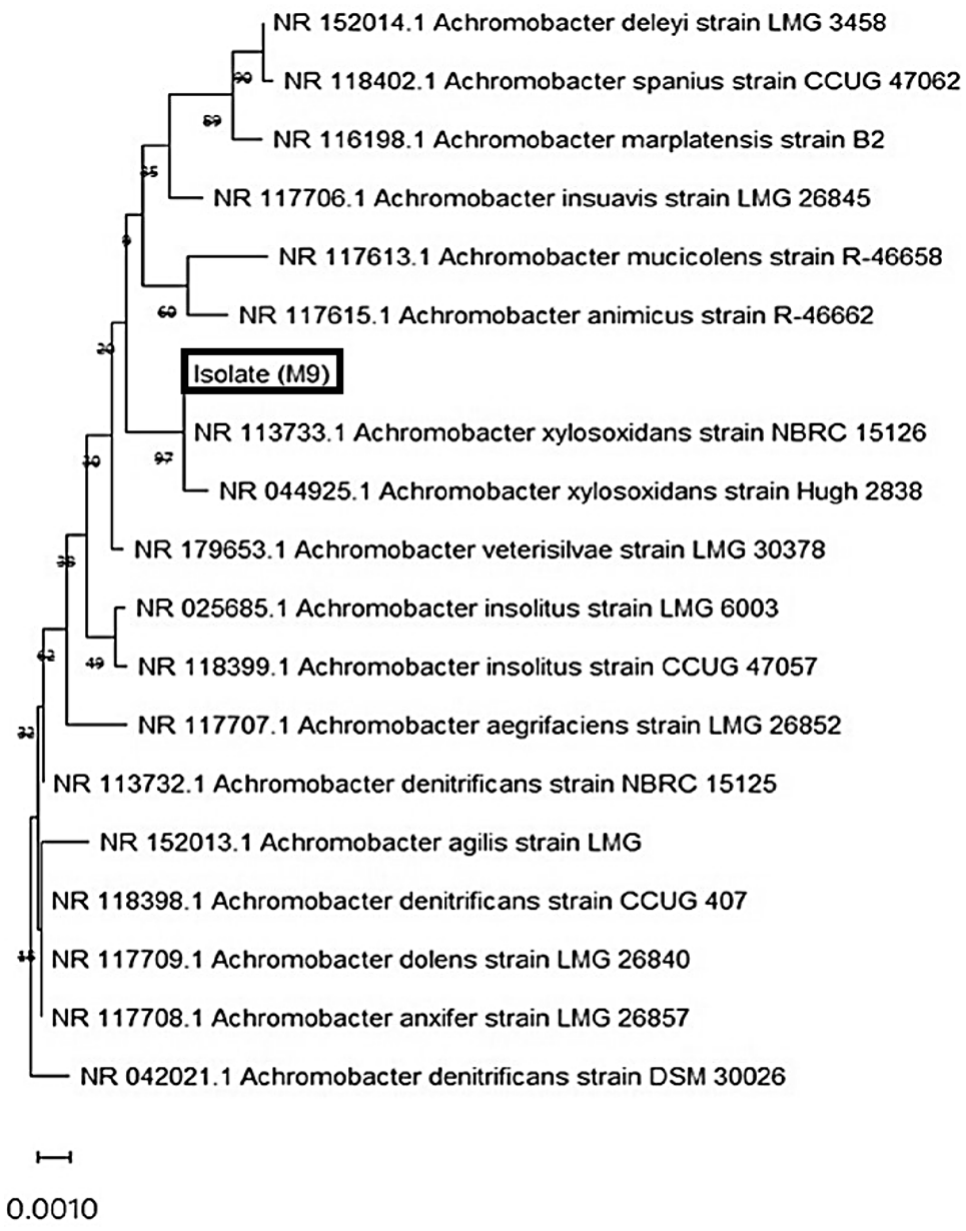
Phylogenetic analysis of isolate *A. xylosoxidans* M9

The promising NPS degraders were identified as *Pseudomonas aeruginosa M2* (PP126564) *Pseudomonas stutzeri M3* (PP126565), *Pseudomonas* sp M7 (PP126566), *Pseudomonas aeruginos*a M8 (PP126567) and *Achromobacter xylosoxidans M9* (OR859752). The phylogenetic tree was constructed to the isolate *Achromobacter xylosoxidans M9* showed 97% evolutionary homology with *Achromobacter* NR113733.1 and NR 044925.1. The nearest relative, *Achromobacter aegrifaciens*, has a 62% similarity. The partial sequence of the 16Sr RNA was deposited to the National Center for Biotechnology Information) NCBI with accession numbers is shown in Fig. 8.

### Particle size and count analysis of NPS

**Fig. 9:**
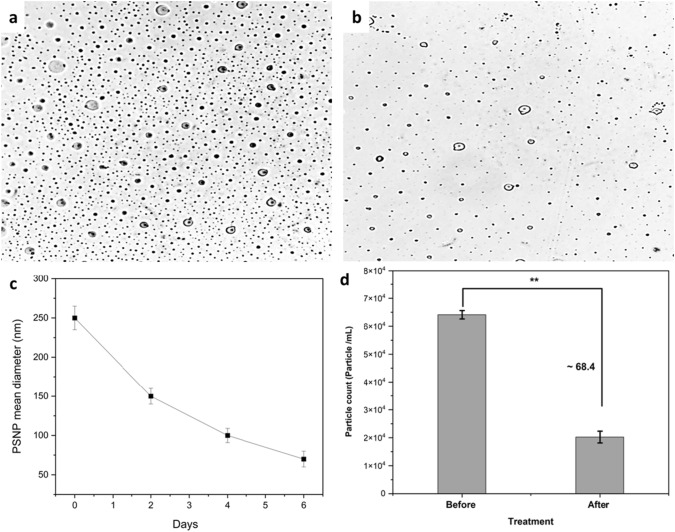
**a** High-contrast scan image of the nanoplastic before treatment with M9 **b** High-contrast scan image of the nanoplastic after treatment **c** Size reduction rate on PSNP after treatment with *Achromobacter xylosoxidans* M9 **d** Bar chart of the nanoplastic numbers before and after treatment

**Fig. 10:**
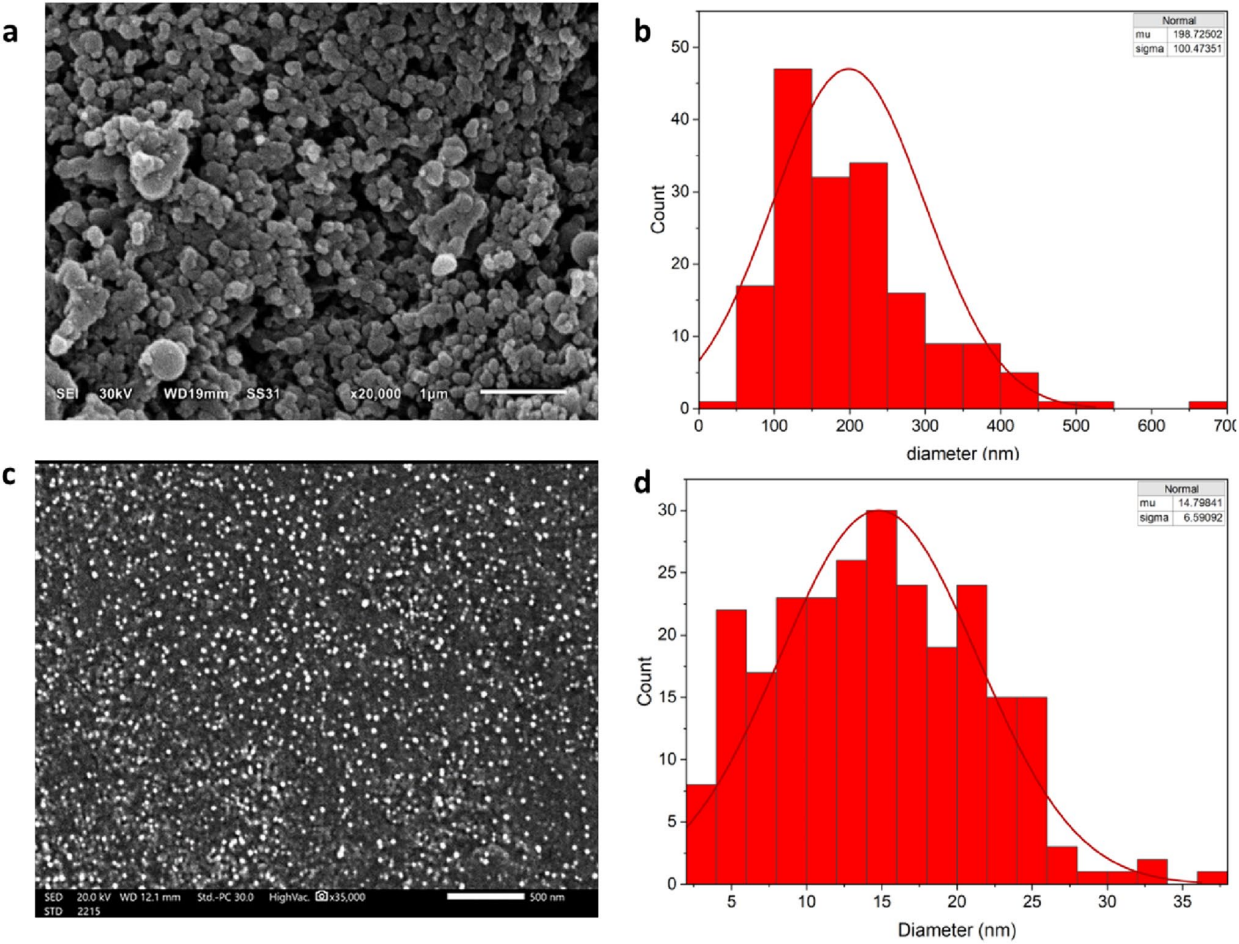
Scanning electron microscope (SEM) image of the NPS **a** before degradation **b** size distribution histogram of NPS before degradation with M9 **c** SEM image after degradation **d** size distribution histogram of NPS after degradation

**Fig. 11:**
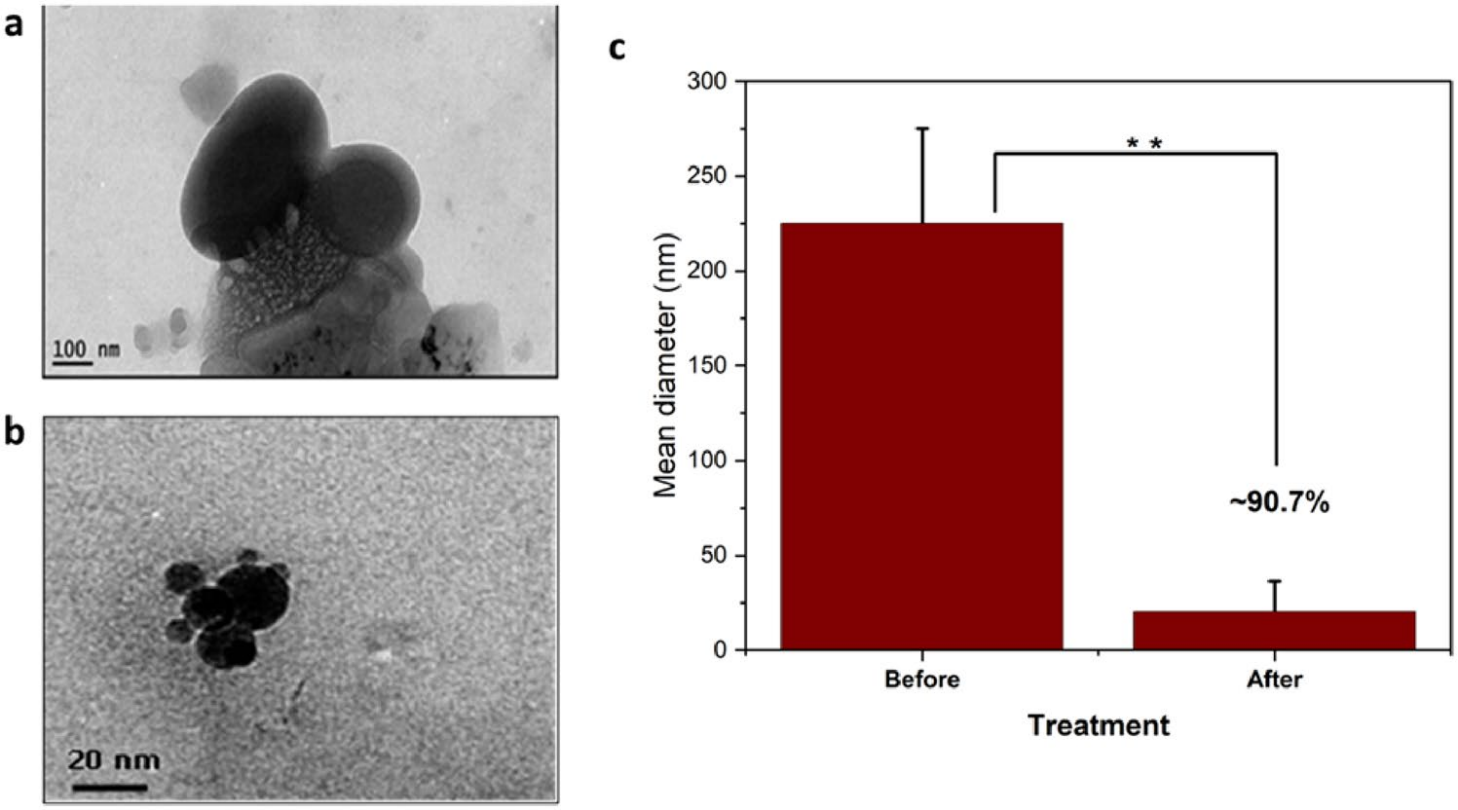
Transmission electron microscope (TEM) image of the NPS **a** before NPS degradation with M9 **b** after degradation **c** percentage of reduction in size. (bars indicate SD, asterisk indicate significance)

Nanoplastic removal efficiency was highlighted through several methodologies and key findings in this study. The nanoplastics were effectively diminished in size through microbial degradation, by *Achromobacter xylosoxidans strain M9*, which demonstrated a significant 68.4% reduction in nanoplastic particle counts within 4 days, as quantified from an initial count of 6.4 × 10^4^ to 2.1 × 10^4^ particles/mL (Fig. 9). Surprisingly, the count increased to 2.9 × 10^4^ particles/mL by the sixth day, however, microscopic analysis (SEM and TEM) revealed a reduction in individual particle size from 0.3 to 0.02 µm or smaller (Fig. 10, 11). This physical shrinkage was corroborated by zeta sizer measurements, indicating a drastic decrease in particle size from 250 ± 10 to 70 ± 15 nm after 6 days, equating to a 73% reduction. These observations underscore the dynamic nature of nanoplastic degradation, emphasizing the critical role of microbial action in altering nanoplastic physical properties over time. This comprehensive approach, combining particle count, dynamic light scattering, and SEM imaging, provides a robust framework for assessing NPS degradation, highlighting the substantial impact of microbial biodegradation on reducing nanoplastic pollution.

### ATR-FTIR analysis of NPS

The ATR-FTIR analysis provided insightful data on the biodegradation of NPS by highlighting changes in functional groups and surface modifications. The analysis revealed two distinct sets of C–H stretches in PS: alkyl C–H bonds ranging from 2800 to 2980 cm^−1^ and aromatic C–H bonds from 3000 to 3125 cm^−1.^ The decrease in intensity of the aromatic peaks with degradation suggests that the primary site of degradation occurs at the benzyl ring of PS, with peaks around 1450 and 1550 cm^−1^ indicating the presence of unconjugated double bonds, confirming attachments to the benzyl ring (Table 5).

**Table 5:** Wavenumbers of the functional groups of the ATR-FTIR analysis (Nandiyanto et al. 2019)

Wavenumber (cm^−1^)	Functional group	NPS	Control	NPS + *A. xylosoxidans*
1000–1300	C–O	Stretching	Stretching	Bending
3230–3550	O–H (Alcohols)	Bending	Bending	Sharp peak, stretching
3040–3010	Terminal (vinyl) C–H stretch	Stretching	Stretching	Attenuation
1510–1450	Aromatic ring stretch	Stretching	Stretching	Sharpening
1600	Conjugated C=C	Stretching	Stretching	Narrowing
1250	Epoxy and oxirane rings	Bending	Bending	Broad bending
720–590	Alcohol, OH out-of-plane bend	Stretching	Stretching	Attenuation
1200	Phenol, C–O stretch	Bending	Bending	Broad bending
1690–1675	Quinone or conjugated ketone	Stretching	Stretching	Narrowing

**Fig. 12:**
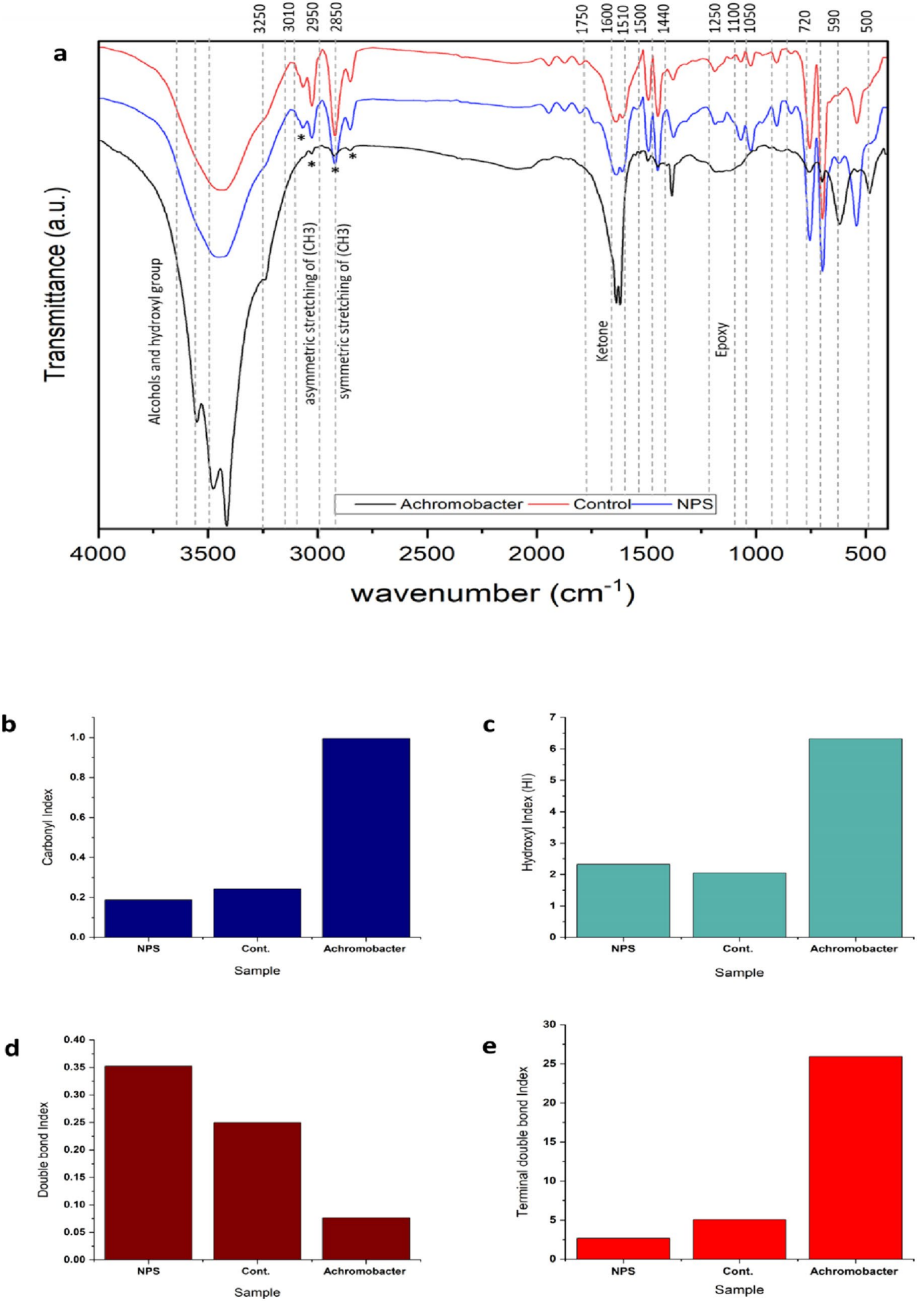
**a** ATR-FTIR graph of the NPS after growth of M9 **b** carbonyl index **c** hydroxyl index **d** double bond index **e** terminal double bond index

Further examination identified the incorporation of alcohol and carboxylic acid functional groups into the styrene framework, as indicated by overlapping O–H vibrations in the 3200–3650 cm^−1^ range and a broad peak below the C–H vibrations, likely representing the –COOH group of carboxylic acids. The significant peak at approximately 1600 cm^−1^ supports the presence of carboxylic acids, suggesting vibrations of the carbonyl (C=O) bond. These findings imply an oxidation process enhancing polystyrene’s surface adhesion and hydrophilicity, likely due to microbial metabolites. Additionally, the detection of spectral bands between 1730 and 1650 cm^−1^ associated with the carbonyl functional group (–C=O) and the presence of ether bonds (–C–O–C–) in the 1150–1075 cm^−1^ range further confirmed the biodegradation of NPS. These alterations suggest the inclusion of oxygen into the polystyrene chain, emphasizing the degradation targeting the aromatic ring and the transformation of the material’s chemical structure through microbial action (Fig. 12).

### Gas chromatography mass spectroscopy

The compound with the highest peak percentage is cis-2,3-Epoxyoctane at 63.59%, detected at a retention time of 1.083 min, indicating it is the most abundant compound in the sample. This is followed by Phenol,2,4-bis (1,1-dimethylethyl)-with a peak percentage of 3.74% at a retention time of 30.057 min, suggesting a significantly lower concentration compared to the leading compound. Other notable compounds include n-Hexadecanoic acid, 1-Dodecanol, 2-octyl-, and Heptadecane, with peak percentages ranging from 1.6 to 1.33%, showing a diverse array of chemical substances within the sample.

Lesser abundant compounds, with peak percentages around or below 1%, include Propyne, 1-Decanol, 2-hexyl-, and Decane, 1-iodo-, among others, highlighting a wide variety of chemical profiles present. The data also reveals the presence of complex compounds such as Diisooctyl phthalate and Octadecanoic acid at later retention times (43.732 and 43.054 min, respectively), suggesting a broad range of volatilities and polarities among the detected chemicals (Table 5).

The comprehensive GC–MS analysis, alongside ATR-FTIR analysis, SEM, DLS, and other methodologies, conclusively demonstrates the biodegradation of polystyrene nanoparticles, marking significant progress in understanding the microbial degradation pathways of plastic pollutants.

### Effect of NPS on biofilm formation

**Fig. 13:**
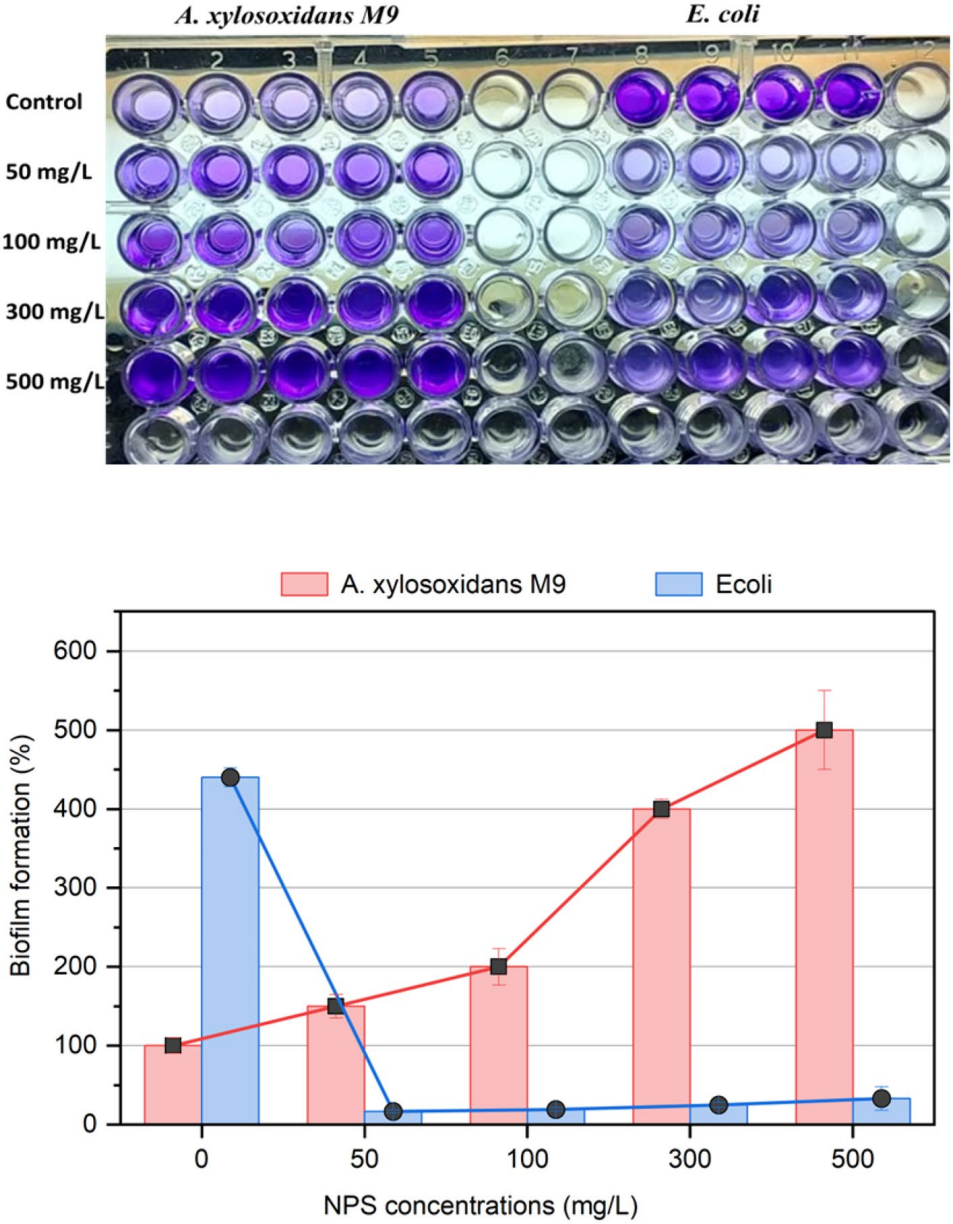
Biofilm behavior of NPS on *A. xylosoxidans* M9 and *E. coli*

The analysis of biofilm formation in response to varying concentrations of nanopolystyrene (NPS) shows that *Achromobacter xylosoxidans M9* exhibited a substantial increase in biofilm production, with a 50% increase at 50 mg/L, doubling at 100 mg/L, 400% at 300 mg/L, and a remarkable fivefold (500%) increase at the highest concentration of 500 mg/L compared to the control. In contrast, *E. coli* demonstrated a marked decrease in biofilm formation with NPS exposure, with reductions of 83.3% at 50 mg/L, 80.8% at 100 mg/L, 75% at 300 mg/L, and 66.7% at 500 mg/L compared to its control group. This dichotomy highlights a strain-specific response to NPS, with M9’s biofilm production being stimulated by higher NPS concentrations, whereas *E. coli’*s biofilm formation is significantly inhibited even at the lowest NPS concentration (Fig. 13).

## Discussion

Numerous investigations into the breakdown of polystyrene styrofoam by larvae from the Coleoptera and Lepidoptera orders have shown that the microorganisms in their gut significantly contribute to the degradation of nanoplastic particles ingested into their digestive tract. However, there is still much to be discovered. This study aims to isolate and identify bacterial strains with the ability to degrade nanopolystyrene. The identification of bacteria capable of degrading nanoplastics will facilitate their use in the bioremediation of nanoplastic pollutants in natural ecosystems.

Our study’s findings align with Lou et al. (2021) investigations in plastic degradation, where differences in survival and pupation rates were reported across various diet groups. Lou et al. (2021) indicated that a deficiency in essential nutrients for the growth and pupation of mealworms led to infertility in those consuming plastics. Similarly, (Brandon et al. (2018) connected the low survival and fertility rates to the nutrient-poor, plastic-only diets that fail to provide the necessary resources for protein production essential for the larvae’s development. Tsochatzis et al. (2021) in a more recent study, undertook metabolomic profiling to understand polystyrene assimilation in the larval gut, uncovering the build-up of the toxic substance, 2,4di-tert butylphenol (2,4-DTBP), in the mealworm tissues and frass. Interestingly, Chen and Dai (2015) explored the cytotoxic effects of 2,4-DTBP on the spider mite *Tetranychus cinnabarinus,* discovering pronounced adulticidal and larvicidal impacts, suggesting that these effects are dose-dependent. Our study supports Tsochatzis et al. (2021) hypothesis, which helps explain the observed decline in pupation rates in worms fed solely on plastic compared to those provided with a nourishing diet.

Biodegradation is influenced by the presence of diverse microorganisms in the environment, with the breakdown of materials being significantly affected by the specific environmental conditions. Our study identified and examined five bacterial strains are suggested to degrade polystyrene, namely *Pseudomonas aeruginosa M2, Pseudomonas stutzeri M3*, *Pseudomonas *sp* M7, Pseudomonas aeruginosa M8,* and *Achromobacter xylosoxidans* M9 isolated from the intestines of yellow mealworms. Prior research has shown that *Pseudomonas aeruginosa* can break down PS and previously isolated from the gut of mealworm larvae (Yang et al. 2015b), and *Achromobacter xylosoxidans* M9 have been observed degrading petroleum hydrocarbons and different types of plastics (Singh and Singh 2011; Pradeep et al. 2015a, b; Kowalczyk et al. 2016). Previous literature showed that the biodegradation pathway of *Achromobacter* involves the breakdown of various hydrocarbon pollutants, including both aliphatic and polycyclic aromatic hydrocarbons (PAHs). *Achromobacter* sp. HZ01, isolated from crude oil-contaminated seawater, demonstrated high degradability of total n-alkanes (up to 96.6%) and could effectively utilize PAHs as its sole carbon source, removing significant amounts of anthracene, phenanthrene, and pyrene. This suggests *Achromobacter’s* potential in bioremediation of environments polluted by crude oil (Deng et al. 2014). A different strain, *Achromobacter* sp. LH-1, demonstrated a degradation capability of 94% for phenanthrene (PHE) and has the ability to break down various polycyclic aromatic hydrocarbons (PAHs), showcasing *Achromobacter’s* adaptability in eliminating hydrophobic pollutants. Furthermore, this research suggested several pathways through which strain LH-1 could decompose PHE (Hou et al. 2018). Research also highlighted *Achromobacter’s* capacity to break down pyrene and high-density polyethylene (HDPE), further illustrating the extensive array of contaminants that *Achromobacter* strains are capable of metabolizing. Specifically, *Achromobacter denitrificans* and *Achromobacter xylosoxidans* have been recognized for their contributions to the degradation of pyrene and HDPE, respectively (Kowalczyk et al. 2016; Mawad et al. 2016). Additionally, whole genome sequence analysis of *Achromobacter* revealed the presence of biodegradation pathway in their genome revealing its potential in xenobiotic degradation (Marzec-Grządziel and Gałązka 2023). This evidence supports the hypothesis this strain is effective in degrading PS.

*Achromobacter* species are known for their wide array of enzymes, which serve various functions in their ecological niches. Studies have identified the production of proteolytic enzymes, lipases, bacteriolytic proteases, cellulases, and beta-lactamases among others (Khan et al. 1967; Miyazawa et al. 2001). These enzymes are not only indicative of the ecological adaptability of *Achromobacter* but also suggest their potential utility in various industrial applications.

To understand the role of enzymes in *Achromobacter* species and their potential industrial applications, it is essential to delve into their enzymatic diversity and mechanisms of action, especially regarding the degradation of environmental pollutants such as nanoplastics. Our study screened enzymatic activity of *Achromobacter xylosoxidans M9*, showed an ability to produce enzymes like laccases, cellulases and esterases. Previous literature explored the enzymatic activity of *Achromobacter* species in environmental applications, particularly in the degradation of plastic pollutants like di(2-ethylhexyl) phthalate (DEHP). Wang et al. (2021) suggested the biodegradation pathway of *Achromobacter* sp. RX to degrade DEHP. Utilizing esterases, a de-esterification process was initiated, resulting in the production of mono-(2-ethylhexyl) phthalate (MEHP). This compound underwent further de-esterification, leading to the formation of phthalic acid and subsequently benzoic acid. This sequence of reactions facilitated a 91.7% mineralization within a span of 96 h. A genomic analysis by Gutierrez-Urrutia et al. (2018) revealed the potential of *Achromobacter xylosoxidans* MT3 in metabolizing nitro-phenolic and phenolic compounds, they also confirmed that MT3 displayed the genetic machinery for the metabolism of chlorocatechols and chloromuconates. Esterases catalyze the hydrolysis of ester bonds present in organic compounds, leading to the formation of an alcohol and an acid.

The formation of acids through esterase activity represents a key step in the microbial degradation of synthetic organic compounds (Yoshida et al. 2016). It signifies the transition of potentially toxic and recalcitrant materials into intermediates that are more susceptible to biodegradation. This process is essential for the natural attenuation of pollutants in the environment and can be harnessed for bioremediation applications. In our investigation, acid production served as a secondary screening method to identify bacteria capable of degrading nano-polystyrene (NPS). This approach was predicated on the hypothesis that acid formation during microbial growth on NPS would indicate the biodegradation of this polymer. *Achromobacter xylosoxidans M9* was found to thrive on NPS as a sole carbon source, leading to the production of acid as an intermediate by-product of the biodegradation process. This outcome not only confirmed the metabolic versatility of this strain but also underscored its potential in the bioremediation of NPS. The lack of acid production on a sucrose medium indicated a substrate-specific response, highlighting the organism’s specialized adaptation or enzymatic machinery tailored for xenobiotic degradation (Marzec-Grządziel and Gałązka 2023).

In this study, we conducted an initial analysis using a range of databases and various keywords related to the biodegradation of polystyrene and *Achromobacter* species isolated from mealworm larvae and/or other environmental sources. The data, as outlined in Table S3, reveal a rareness of studies focusing specifically on the role of *Achromobacter* in polystyrene degradation. This scarcity of research underscores the novelty and potential significance of exploring *Achromobacter’s* capacity for polystyrene (PS) biodegradation, considering the organism’s known robust enzymatic activity and historical efficacy in xenobiotic degradation (Table S2). In addition, the absence of detailed investigations into the genetic support and metabolic pathways, such as those annotated in the Kyoto Encyclopedia of Genes and Genomes (KEGG) database, signifies a critical gap in our understanding of PS degradation by this strain. This gap underscores a significant opportunity for future research to elucidate the molecular mechanisms and specific genetic elements that facilitate PS degradation. Without comprehensive gene identification and KEGG pathway analysis, the biochemical processes and enzymatic activities driving PS biodegradation remain speculative. Addressing this gap could substantially advance our knowledge of the metabolic capacities required for PS degradation, potentially unveiling novel strategies for bioremediation and sustainable waste management.

Our research sought to comprehend the mechanism behind the biodegradation of polystyrene by *A. xylosoxidans M9* and aimed to validate the theory that the biodegradation efficiency is related to the size of the polystyrene (Table 6).

**Table 6:** The compounds produced during the degradation of polystyrene (GCMS)

Peak	R. Time (min)	Peak (%)	Compound name	Common name	Industerial applications	Formula	MW
1	1.083	63.59	Cis-2,3-Epoxyoctane	ND	ND	C8H16O	128
**23**	**30.057**	**3.74**	**Phenol, 2,4-bis(1,1-dimethylethyl)-**	**2,4-DTBP**	**Pesticide, Antifungal, Antioxidant**	**C14H22O**	**206**
24	39.969	1.6	n-Hexadecanoic acid	Palmitic acid	Soap and Detergent	C16H32O2	256
20	24.968	1.4	1-Dodecanol, 2-octyl-	2-Octyldodecanol	Skin care and cosmetics	C20H42O	298
5	11.058	1.33	Heptadecane	ND	ND	C17H36	240
3	1.288	1.06	Propyne	ND	ND	C4H10N2	40
10	15.913	1	1-Decanol, 2-hexyl-	2-Hexyldecanol	ND	C16H34O	242
16	21.08	0.79	Decane, 1-iodo-	decyl iodide	Key intermediate in diverse chemical syntheses	C10H21I	268.1
19	24.759	0.69	Tetracosane	ND	Wax industry	C24H50	338
17	21.442	0.6	Heptasiloxane, hexadecamethyl-	ND	Medical device coatings	[(CH3)2SiO]7	532
12	20.633	0.57	Heptadecyl heptafluorobutyrate	Heptadecyl heptafluorobutanoate	ND	C21H35F7O2	452
4	10.799	0.56	Nonadecane	ND	ND	C19H40	268
7	11.946	0.51	Cyclohexane, undecyl-	ND	ND	C17H34	238
14	20.908	0.5	5,5-Diethylheptadecane	ND	ND	C21H44	296
13	20.707	0.49	Hexadecane, 2,6,10,14-tetramethyl-	Phytane	Geomarker for petroleum compounds	C20H42	282.5475
18	23.173	0.49	Decane, 1,1′-oxybis-	Dioctyl Ether	Lubricant	(C8H17)2O	298
26	43.732	0.44	Diisooctyl phthalate	DIOP	Plasticizer	C24H38O4	390
25	43.054	0.42	Octadecanoic acid	Stearic acid	Emulsifier	C18H36O2	284
11	16.5	0.38	Eicosane, 2,4-dimethyl-	2,4-Dimethylicosane	INSECT HYDROCARBONS	C22H46	310
21	28.676	0.38	Hexatriacontane	ND	Wax industry	C36H74	506
15	15.764	0.36	Eicosane	Icosane	ND	C20H42	282.5
22	29.201	0.33	Dotriacontane	ND	Lubricants, in paraffin waxes	C32H66	450
8	12.15	0.31	Dodecane, 4,6-dimethyl-	ND	ND	C14H30	198
6	11.207	0.3	Hexadecane	ND	ND	C16H34	226

Bold values indicate higher values and the most important components in the table

In the previous research, it was observed that the breakdown of nanopolystyrene emulsion occurs at a quicker and more effective rate compared to the biodegradation of polystyrene film (Mohan et al. 2016). Also, the biodegradation of the microplastic beads needed an average 20–60 days to show a degradation rate less than 7% (Table 7). Our study is compatible with the previous research, the biodegradation rate of NPS particles was ~ 68% in 4 days, however the PS film showed only 7% weight loss in 30 days. Thes observations can be explained by several factors intrinsic to the physical properties and surface area of the materials. NPS, with its significantly smaller particle size, presents a much larger surface area relative to its volume when compared to bulkier PS forms, such as films or beads. This increased surface area allows for greater microbial access and attachment, facilitating the enzymatic degradation process. Also, it was also suggested that the biofilm formation is depending on the concentration of the nanoplastic released in the media.

**Table 7:** List of symbiotic bacteria with the ability to degrade polystyrene

Organism	Isolation source	PS form/size	Incubation (days)	Biodegradation/weight loss (%)	References
*Achromobacter xylosoxidans M9*	*Tenebrio molitor*	NPS, PS film	30	7	This study
*Pseudomonas aeruginosa M2*	*Tenebrio molitor*	NPS, PS film	30	6	This study
*Acinetobacter septicus* *Agrobacterium tumefaciens* *Klebsiella grimontii* *Pseudomonas multiresinivorans* *Pseudomonas nitroreducens* *Pseudomonas plecoglossicida* *Serratia marcescens* *Yokenella regensburgei*	*Tenebrio molitor*	Micro-sprayed, PS-film	14	Growth on agar and cracks in PS film	Park et al. (2023)
*Klebsiella oxytoca*	*Tenebrio molitor*	EPS Emulsion	ND	ND	Machona et al. (2022)
*Klebsiella *sp*. WJ2020*	*Tenebrio molitor*	PS film	60	4.35	Lin et al. (2024)
*Cellulosimicrobium *sp*. WJ2025*	*Tenebrio molitor*	PS film	60	6.93	Lin et al. (2024)
*Exiguobacterium *sp*. YT2*	*Tenebrio molitor*	PS film	30	7.4 ± 0.4	Yang et al. (2015a, b)
*Masilia *sp*. FS1903*	*Galleria mellonella*	PS film	30	12.97 ± 1.05	Jiang et al. (2021)
*Serratia *sp*. WSW*	*Plesiophthalmus davidis*	PS film	20	Crakes in the PS film and biofilm formation	Woo et al. (2020)
*Pseudomonas aeruginosa DSM 50071*	*Zophobas atratus*	PS film	ND	confirmed by the SH inhibitor treatment test	Kim et al. (2020)
*Priestia megaterium S1*	*Tenebrio molitor*	Beads	180	28.6	Akash et al. (2023)
*Enterobacter hormaechei*	*Tenebrio molitor*	NPS, PS film	15	2.76 ± 0.22 (anaerobic)	Kang et al. (2023)
*Serratia marcescens*	*Tenebrio molitor*	PS Emulsion		Microbiome study	Urbanek et al. (2020)
*Klebsiella oxytoca*	*Tenebrio molitor*	PS Emulsion		Microbiome study	Urbanek et al. (2020)
*Acinetobacter *sp*. AnTc-1*	*Tribolium castaneunm*	PS powder, film	60	12.14	Wang et al. (2020)
*Bacillus *sp.	*Galleria mellonella*	Microbiome study			Lou et al. (2021)
*Enterococcus *sp.	*Tenebrio molitor, Galleria mellonella, and Zophobas atratus*	Microbiome study			Jiang et al. (2021)
*Serratia *sp*.*	*Tenebrio molitor*	PS styrofoam	Microbiome study		Mamtimin et al. (2023)
*Staphylococcus *sp*.*	*Tenebrio molitor*	PS styrofoam	Microbiome study		Mamtimin et al. (2023)
*Rhodococcus *sp.	*Tenebrio molitor*	PS styrofoam	Microbiome study		Mamtimin et al. (2023)
*Pseudomonas aeruginosa*	*Tenebrio molitor*	PS (raw material, processed and expanded polystyrene, and polystyrene used for packaging)	Microbiome study		Urbanek et al. (2020)

The release of NPS particles from polystyrene film into aqueous media (Fig. S2) can serve as a stimulant for biofilm formation on the polystyrene surface. This phenomenon suggests a relationship between the presence of NPS particles and the facilitation of biofilm development. Our findings indicate that biofilm formation on the polystyrene film surface is positively correlated with the concentration of NPS in the medium. As the concentration of NPS increases, there is a corresponding increase in biofilm formation. On the contrary, NPS can inhabit the biofilm formation and growth of other microbes such as *E. coli*. This interesting mechanism needs further studies. This relationship highlights the dual role of NPS particles: they not only alter the physical integrity of the polystyrene surface, evidenced by the emergence of cracks and holes, but also promote the initial stages of microbial interaction necessary for biodegradation. The enhancement of biofilm formation by NPS particles suggested a complex interplay between the physical degradation of the polystyrene material and biological degradation processes. Understanding this mechanism in greater detail could reveal significant insights into the biodegradation of synthetic polymers and inform strategies to mitigate the environmental impact of plastic waste.

The ATR-FTIR and GC–MS analyses shed light on the biodegradation mechanisms of polystyrene nanoparticles (NPS), providing a comprehensive understanding of the chemical and microbial pathways involved in the breakdown of this persistent pollutant. The ATR-FTIR data reveal critical changes in the chemical structure of NPS, most notably through the identification of altered functional groups that mark the biodegradation process. A significant finding from the FTIR analysis is the decrease in aromatic C–H bond intensities, which indicates that the benzyl rings of the polystyrene are the primary sites of microbial attack. This is complemented by the appearance of alcohol and carboxylic acid functional groups, signifying the oxidation of the polystyrene structure that enhances its hydrophilicity and surface adhesion properties. These transformations suggest not only the inclusion of oxygen into the polystyrene chain but also a targeted degradation process that focuses on the aromatic components, facilitating the material’s breakdown.

The discovery of 2,4-di-tert-butylphenol (2,4-DTBP) in the GC–MS analysis of polystyrene nanoparticles (NPS) biodegraded within mealworm larvae aligns with findings previously reported by Tsochatzis et al. (2021), offering significant insights into the internal degradation processes of these organisms. This particular compound’s presence is intriguing as it points towards the biochemical pathway mealworms employ to decompose plastic materials, specifically NPS, which are notoriously resistant to environmental breakdown. 2,4-DTBP is known for its high persistence and bioaccumulation potential, raising interesting questions about its role and implications in the biodegradation process. The presence of 2,4-DTBP as a degradation product underscores the complexity of biodegradation processes and the intricate relationship between mealworms and their gut microbiota. It also opens up avenues for further research into harnessing these biological systems for environmental cleanup efforts, particularly in breaking down persistent plastic pollutants. However, it also calls for a careful assessment of the degradation products formed, their environmental impact, and potential accumulation in food chains, underscoring the need for a comprehensive understanding of biodegradation processes before their application in mitigating plastic pollution can be fully realized.

The biodegradation pathway of polystyrene by *Achromobacter xylosoxidans M9* elucidates a complex microbial mechanism that converts a recalcitrant synthetic polymer into a variety of smaller, environmentally benign compounds. The initial step in this pathway is the oxidative cleavage of the polystyrene polymer, primarily facilitated by enzymes such as oxygenases. These enzymes introduce oxygen into the polymer backbone, leading to the formation of smaller aromatic intermediates through the breaking of carbon–carbon bonds. This critical step underscores the role of oxidative enzymes in initiating the breakdown of the highly stable polystyrene polymer. Following the initial cleavage, the pathway diverges into the enzymatic transformation of these aromatic intermediates into even smaller aromatic compounds, such as various substituted phenols (e.g., 2,4-DTBP). This transformation involves additional enzymatic actions, including further oxidation, which rearranges the molecular structure of the intermediates. Such enzymatic processes are pivotal in breaking down complex aromatic structures into compounds that are more amenable to microbial metabolism. As the degradation process progresses, a significant transformation occurs from aromatic to aliphatic compounds. This step is mediated by a suite of enzymes, including those responsible for ring-hydroxylating and ring-cleaving dioxygenases. These enzymes are capable of opening aromatic rings, a necessary process for the conversion of aromatic molecules into aliphatic chains. The products of these reactions include a variety of aliphatic acids, alcohols, and hydrocarbons, illustrating the diversity of microbial enzymatic capabilities in processing polymer degradation products. The detection of specific compounds such as cis-2,3-Epoxyoctane and Heptadecylheptafluorobutyrate among the degradation products highlights the pathway’s complexity and the broad enzymatic repertoire of *Achromobacter xylosoxidans M9*. These compounds indicate not only the primary enzymatic actions involved in the degradation of polystyrene but also secondary reactions that further modify the breakdown products. Such secondary modifications can include epoxidation, fluorination, and hydroxylation, which are indicative of the microorganism’s ability to adapt its enzymatic profile to the chemical structure of the degradation intermediates.

## Conclusions

The major bacterial community identified in this investigation consisted mostly of the Proteobacteria phylum. The 16S ribosomal RNA gene sequencing indicated that the five *T. molitor* larval gut isolates belonged to the Pseudomonas and Achromobacter species. Strain Achromobacter xylosoxidans OR859752 was shown to decrease the size of nanopolystyrene particles by 92.3% and alter the morphological properties of the polystyrene film. The results indicate that the intestinal tract of T. molitor is a very effective reservoir of microorganisms with the ability to degrade nanopolystyrene through biological processes. However, further research is required to examine the transcriptome profile of each isolate and its potential uses in biotechnology.

### Supplementary Information

Below is the link to the electronic supplementary material.

Supplementary file 1 (DOCX 4692 kb)

## Data Availability

The data generated and/or analysed during the current study are available from the corresponding author on reasonable request.

## Abbreviations

ATR-FTIR: Attenuated total reflectance-Fourier Transform Infrared Spectroscopy
BCP: Bromocresol purple
CFBM: Carbon-Free Basal Media
DLS: Dynamic light scattering
E. coli: *Escherichia coli*
EPS: Expanded polystyrene
LDPE: Low density polyethylene
MP: Microplastic
NP: Nanoplastic
NPS: Nano-polystyrene
NPS-CFBM: Nano-Polystyrene Carbon-Free Basal Media
OD: Optical density
PS: Polystyrene
PSF: Polystyrene film
SEM: Scanning electron microscope
TEM: Transmission electron microscope
