# Supplementary material for: Biodegradation of polystyrene nanoplastics by Achromobacter xylosoxidans M9 offers a mealworm gut-derived solution for plastic pollution
Source: Arch Microbiol. 2024 Apr 30;206(5):238. doi: 10.1007/s00203-024-03947-z (PMC11058615; doi:10.1007/s00203-024-03947-z)
Supplement: Supplementary file 1 — (DOCX 4692 kb) [file 203_2024_3947_MOESM1_ESM.docx]

**Supporting Information**

**Biodegradation of Polystyrene Nanoplastics by *Achromobacter xylosoxidans* M9 offers a Mealworm Gut-Derived Solution for Plastic Pollution**

Najat El-Kurdi^a,b *^, Mohamed Ghazy^a,c^, , Sherif Hammad^d^, Khaled ElBaghdady^e^ , Sahar El-Shatoury^f*^

^a^ Biotechnology Program, Basic and Applied Science Institute, Egypt-Japan University of Science and Technology, New Burj Al-Arab, Alexandria, Egypt.

^b^ Aquaculture Biotechnology Department, Fish Farming and Technology Institute, Suez Canal University, Ismailia, Egypt.

^c^ Biochemistry Department, Faculty of Science, Ain Shams University, Cairo, Egypt.

^d^ PharmD program, Basic and Applied Science Institute, Egypt-Japan University of Science and Technology, New Burj Al-Arab, Alexandria, Egypt.

^e^ Botany and Microbiology Department, Faculty of Science, Ain Shams University, Cairo, Egypt.

^f^ Microbiology Department, Faculty of Science, Suez Canal University, Ismailia, Egypt.

Authors for correspondence:

sahar_hassan@science.suez.edu.eg **(Prof. Sahar A. El-Shatoury).**

nagat.elkurdi.fish@suez.edu.eg **(Najat El-Kurdi).**


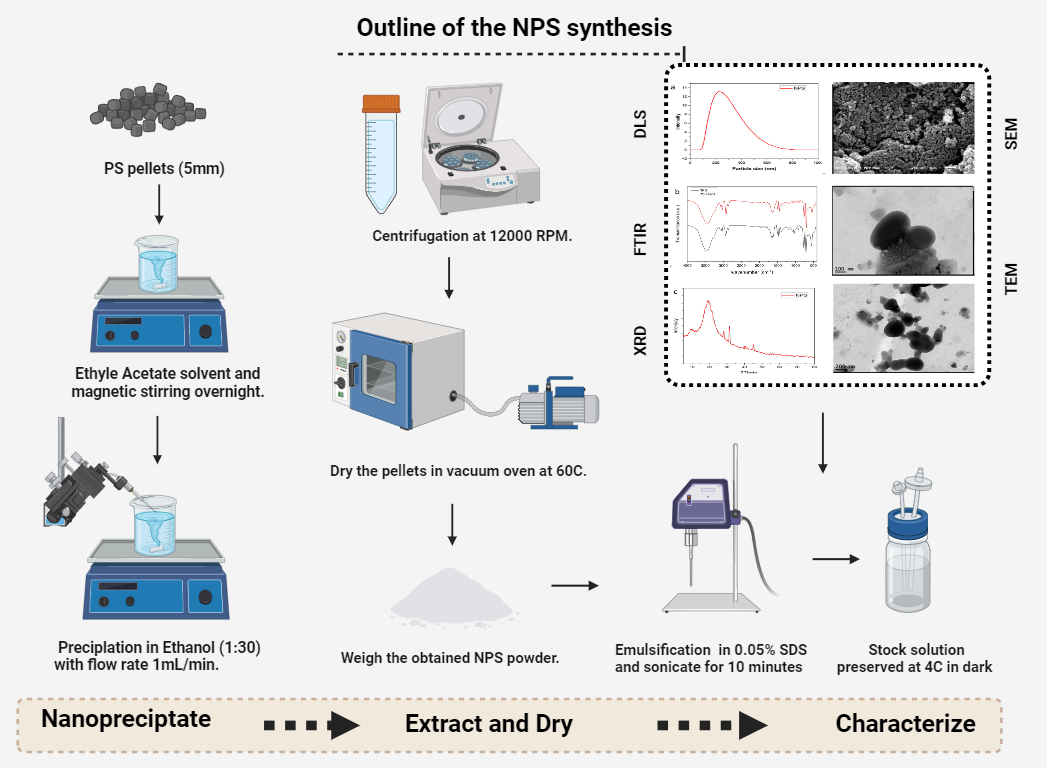

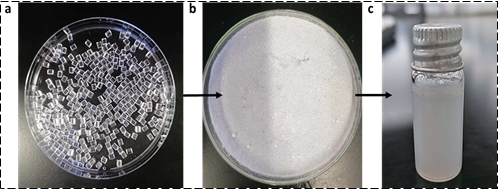


**Fig S1.** Overview of the environmental nanoplastics (NPS) preparation process, illustrating the transition from (a) polystyrene pellets (b) NPS powder, and (c) NPS emulsion (generated by Biorender.com)


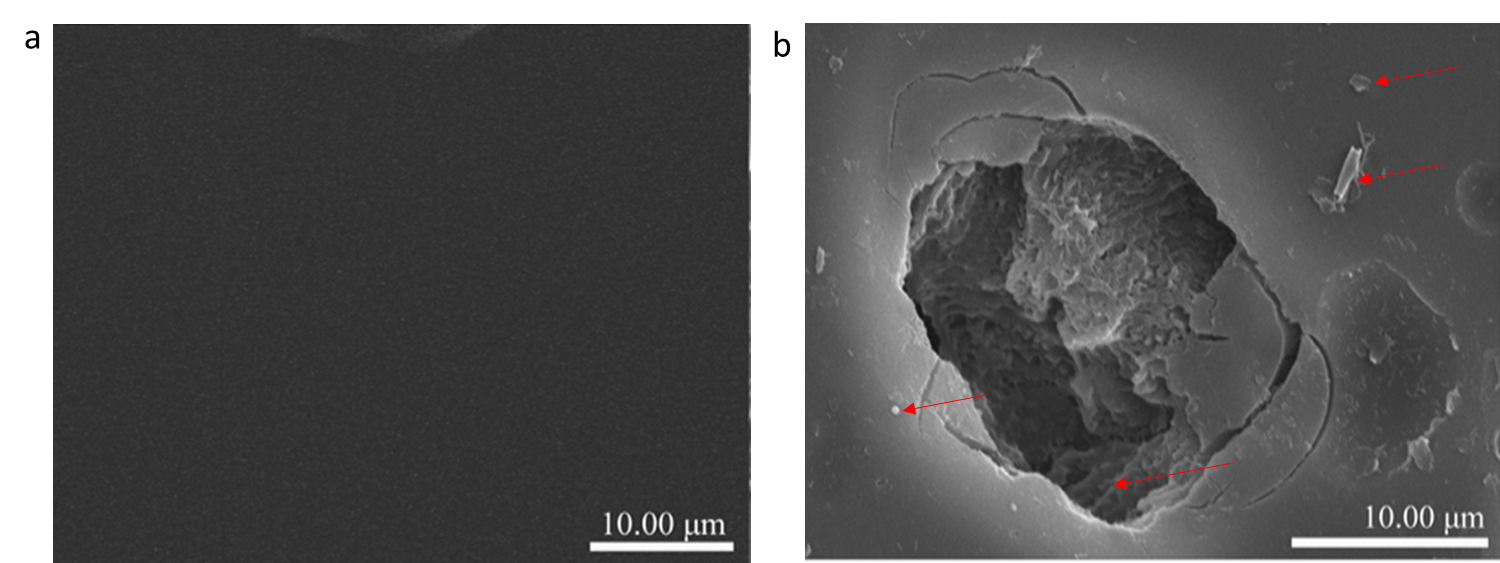


**Fig S2**. Release of the nano and micro-scale plastics from the PS film surface (Z. Wang et al., 2020)


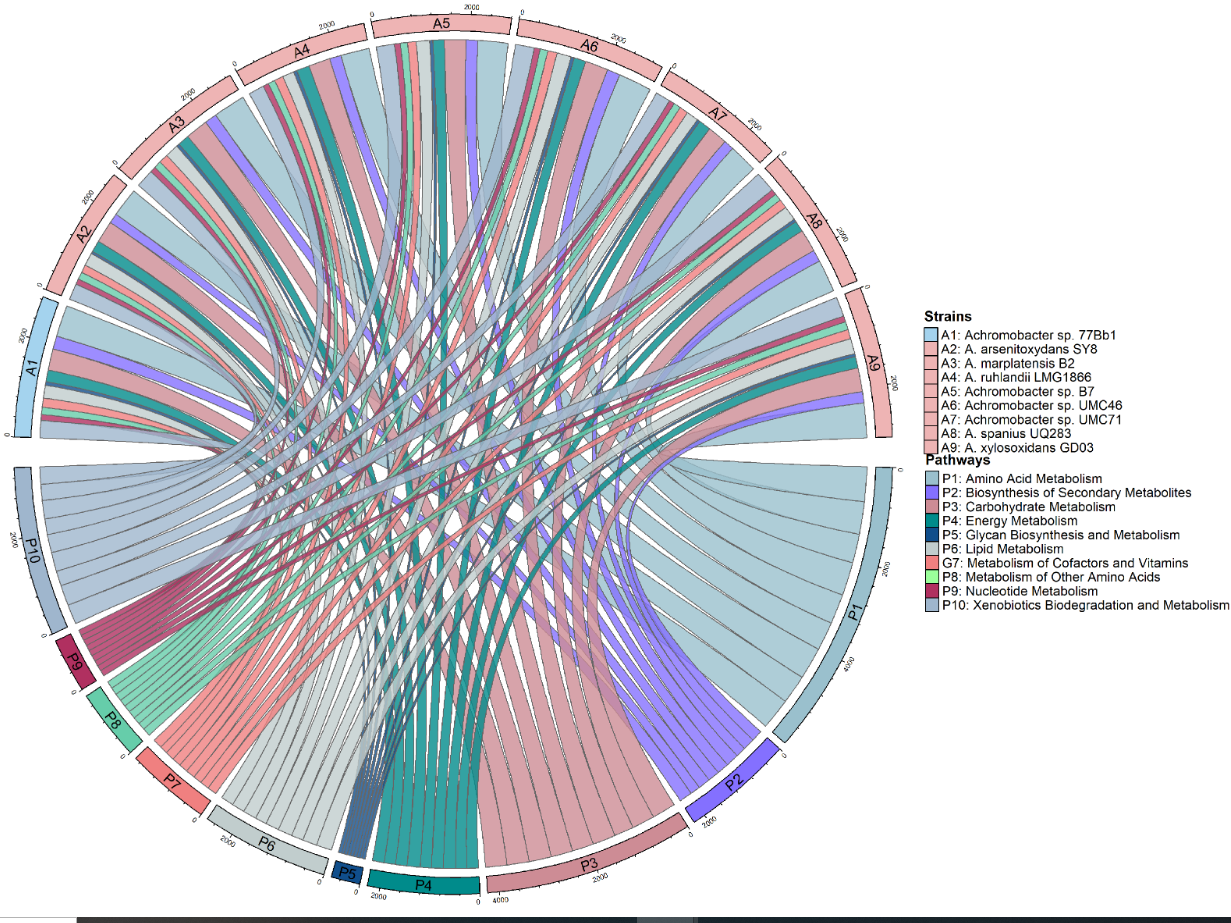


**Fig. S3** Xenobiotic biodegradation pathway among the genome of *A. xylosoxidans* (Marzec-Grządziel & Gałązka, 2023)

| **Table S1** Pollutants degraded by genus *Achromobacter* sp. | | | | |
| --- | --- | --- | --- | --- |
| **Pollutant** | **Species** | **Origin** | **Reference** |  |
| Neonicotinoids | *Achromobacter sp.* | Soil | (Gao et al., 2021) |  |
| Dibutyl phthalate | *Achromobacter pulmonis sp.* | Plastic contaminated water | (Qu et al., 2024) |  |
| Oxytetracycline | *Achromobacter sp. ONK* | Activated sludge | (Nguyen et al., 2023) |  |
| Quinoline | *Achromobacter sp. strain JWJ-09* | Coking wastewater | (Deng et al., 2023) |  |
| Polyaromatic hydrocarbons (PAHs) | *Achromobacter xylosoxidans BP1* | Contaminated soil | (Zhang et al., 2023) |  |
| Terpene | *Achromobacter sp. UMC46* | Soil | (Marzec-Grządziel & Gałązka, 2023) |  |
| Arsenite | *Achromobacter arsenitoxydans SY8.* | Polychlorinated Biphenyl-contaminated soil | (X. Li et al., 2012) |  |
| Phenanthrene | *Achromobacter sp. LH-1* | Soil | (Zang et al., 2020) |  |
| Anthracene | *Achromobacter xylosoxidans strain BUK_BTEG6* | Petrochemical contaminated Soil | (Usman et al., 2023) |  |
| High-density polyethylene | *Achromobacter xylosoxidans* | Soil | (Kowalczyk et al., 2016) |  |
| Di-(2-ethylhexyl) phthalate | *Achromobacter* sp. RX | Activated sludge | (Wang et al., 2021) |  |
| Low-density polyethylene (LDPE) and polyvinyl chloride (PVC) microplastics | *Achromobacter denitrificans* Ebl13 | PVC and LDPE enriched compost sample | (Maleki Rad et al., 2022) |  |
| Pyrene | *Achromobacter sp AC15* | Mangrove soil | (Li et al., 2020) |  |
| Phenanthrene | *Achromobacter sp. PHED2* | Sludge | (Li et al., 2021) |  |
| Sulfamethoxazole (SMX) | *Achromobacter JL9* | Sediments of a pharmaceutical wastewater treatment plant. | (Liang et al., 2021) |  |

| **Table S2.** Main reasons for choosing *Tenebrio molitor* in this study. | | |
| --- | --- | --- |
| **Application** | **Comments** | **Reference** |
| Aquaculture Feed | a high-quality natural protein source, potentially replacing fishmeal in aquaculture diets. | (Hong et al., 2020) |
| Waste Management | *Tenebrio molitor* larvae can biodegrade polyether polyurethan, polystyrene, polyethylene, and polyvinyl chloride suggesting their potential in waste management. | (Brandon et al., 2018; Liu et al., 2022; B. Peng et al., 2023; B.-Y. Peng et al., 2020; Tsochatzis et al., 2021) |
| Organic Waste Recycling | *T. molitor* larvae can be used as a protein source for broiler chickens, recycling organic wastes and showing no significant differences in feed intake, weight gain, or feed efficiency after 15 days. | (Ramos-Elorduy et al., 2002) |
| Nutritional Value for Aquaculture | High content of proteins, fats, and essential amino acids, making *T. molitor* a suitable feed ingredient for fish and other aquaculture species. | (Hua, 2021) |

| **Table S3** Results of the analysis | | | | |
| --- | --- | --- | --- | --- |
| **Keywords** | **Web of science** | **PubMed** | **Google Scholar** | **Scopus** |
| Biodegradation of Polystyrene | 769 | 9,465 | 122,000 | 1,021 |
| Mealworms Plastic Degradation | 57 | 79 | 5,270 | 51 |
| *Tenebrio molitor* Polystyrene | 103 | 64 | 1,800 | 93 |
| Mealworm Gut Microbiota and Polystyrene | 19 | 30 | 1,620 | 16 |
| *Achromobacter* and polystyrene | 1 | 1 | 1 | 0 |
| *Achromobacter* and polystyrene and mealworm | 0 | 0 | 0 | 0 |

**References**

Brandon, A., Gao, S., Tian, R., Ning, D., Yang, S., Zhou, J., Wu, W., & Criddle, C. (2018). Biodegradation of Polyethylene and Plastic Mixtures in Mealworms (Larvae of *Tenebrio molitor*) and Effects on the Gut Microbiome. Environ sci tech, 52(11), 6526–6533. https://doi.org/10.1021/acs.est.8b02301

Deng, M., Yu, F., Wang, J., Yu, J., & Jin, W. (2023). Bio-augmentation effect of *Achromobacter* sp. Strain JWJ-09 on quinoline and real coking wastewater under methanol co-metabolism. J. Water Pro Eng 53, 103611. https://doi.org/10.1016/j.jwpe.2023.103611

Gao, Y., Liu, M., Zhao, X., Zhang, X., & Zhou, F. (2021). Paracoccus and *Achromobacter* bacteria contribute to rapid biodegradation of imidacloprid in soils. Ecotoxicol. Environ. Saf. 225,112785. https://doi.org/10.1016/j.ecoenv.2021.112785

Hong, J., Han, T., & Kim, Y. Y. (2020). Mealworm (*Tenebrio* *molitor* Larvae) as an Alternative Protein Source for Monogastric Animal: A Review. Animals, 10(11), Article 11. https://doi.org/10.3390/ani10112068

Hua, K. (2021). A meta-analysis of the effects of replacing fish meals with insect meals on growth performance of fish. Aquaculture, 530, 735732. https://doi.org/10.1016/j.aquaculture.2020.735732

Kowalczyk, A., Chyc, M., Ryszka, P., & Latowski, D. (2016). *Achromobacter* *xylosoxidans* as a new microorganism strain colonizing high-density polyethylene as a key step to its biodegradation. Environ Sci Poll. Res, 23(11), 11349–11356. https://doi.org/10.1007/s11356-016-6563-y

Li, J., Wang, Y., Zhou, W., Chen, W., Deng, M., & Zhou, S. (2020). Characterization of a new biosurfactant produced by an effective pyrene-degrading *Achromobacter* species strain AC15. Int. Biodeterior. Biodegrad., 152, 104959. https://doi.org/10.1016/j.ibiod.2020.104959

Li, X., Hu, Y., Gong, J., Lin, Y., Johnstone, L., Rensing, C., & Wang, G. (2012). Genome Sequence of the Highly Efficient Arsenite-Oxidizing Bacterium *Achromobacter arsenitoxydans* SY8. J. Bact, 194(5), 1243–1244. https://doi.org/10.1128/jb.06667-11

Li, X., Peng, D., Zhang, Y., Ju, D., & Guan, C. (2021). *Achromobacter* sp. PHED2 enhances the phenanthrene degradation and stress tolerance in maize involving the participation of salicylic acid. Environ. Technol. Innov, 21, 101365. https://doi.org/10.1016/j.eti.2021.101365

Liang, D. hui, Hu, Y., Liang, D., Chenga, J., & Chena, Y. (2021). Bioaugmentation of Moving Bed Biofilm Reactor (MBBR) with *Achromobacter* JL9 for enhanced sulfamethoxazole (SMX) degradation in aquaculture wastewater. Ecotoxicol Environ. Saf., 207, 111258. https://doi.org/10.1016/j.ecoenv.2020.111258

Liu, J., Liu, J., Xu, B., Xu, A., Cao, S., Wei, R., Zhou, J., Jiang, M., & Dong, W. (2022). Biodegradation of polyether-polyurethane foam in yellow mealworms (*Tenebrio* *molitor*) and effects on the gut microbiome. Chemosphere, 304. https://doi.org/10.1016/j.chemosphere.2022.135263

Maleki Rad, M., Moghimi, H., & Azin, E. (2022). Biodegradation of thermo-oxidative pretreated low-density polyethylene (LDPE) and polyvinyl chloride (PVC) microplastics by *Achromobacter denitrificans* Ebl13. Mar. Poll. Bull., 181, 113830. https://doi.org/10.1016/j.marpolbul.2022.113830

Marzec-Grządziel, A., & Gałązka, A. (2023). Sequencing of the Whole Genome of a Bacterium of the Genus *Achromobacter* Reveals Its Potential for Xenobiotics Biodegradation. Agriculture, 13(8), Article 8. https://doi.org/10.3390/agriculture13081519

Nguyen, H. T., Siddiqui, S. I., Maeng, S. K., & Oh, S. (2023). Biological detoxification of oxytetracycline using *Achromobacter*-immobilized bioremediation system. J. Water Pro. Eng., 52, 103491. https://doi.org/10.1016/j.jwpe.2023.103491

Peng, B., Xu, Y., Sun, Y., Xiao, S., Sun, J., Shen, Z., Chen, J., Zhou, X., & Zhang, Y. (2023). Biodegradation of polyethylene (PE) microplastics by mealworm larvae: Physiological responses, oxidative stress, and residual plastic particles. J. Cle. Pro., 402. https://doi.org/10.1016/j.jclepro.2023.136831.

Peng, B.-Y., Chen, Z., Chen, J., Yu, H., Zhou, X., Criddle, C. S., Wu, W.-M., & Zhang, Y. (2020). Biodegradation of Polyvinyl Chloride (PVC) in *Tenebrio molitor* (Coleoptera: *Tenebrionidae*) larvae. Environ. Int., 145, 106106. https://doi.org/10.1016/j.envint.2020.106106

Qu, Y., Chen, J., Russel, M., Huang, W., Bingke, Y., lei, W., Zhang, D., & Blaszczak-Boxe, C. (2024). Optimizing concentration and interaction mechanism of *Demodesmus* sp. And *Achromobacter* *pulmonis* sp. Consortium to evaluate their potential for dibutyl phthalate removal from synthetic wastewater. Bio. Technol., 395, 130372. https://doi.org/10.1016/j.biortech.2024.130372

Ramos-Elorduy, J., González, E. A., Hernández, A. R., & Pino, J. M. (2002). Use of Tenebrio molitor (Coleoptera: *Tenebrionidae*) to Recycle Organic Wastes and as Feed for Broiler Chickens. J. Eco. Ent., 95(1), 214–220. https://doi.org/10.1603/0022-0493-95.1.214

Tsochatzis, E. D., Berggreen, I. E., Nørgaard, J. V., Theodoridis, G., & Dalsgaard, T. K. (2021). Biodegradation of expanded polystyrene by mealworm larvae under different feeding strategies evaluated by metabolic profiling using GC-TOF-MS. Chemosphere, 281, 130840. https://doi.org/10.1016/j.chemosphere.2021.130840

Usman, S., Yakasai, H. M., Gimba, M. Y., Shehu, D., & Jagaba, A. H. (2023). Anthracene degradation by *Achromobacter xylosoxidans* strain BUK_BTEG6 isolated from petrochemical contaminated soil. Case Stu. Chem. Environ. Eng., 8, 100418. https://doi.org/10.1016/j.cscee.2023.100418

Wang, P., Gao, J., Zhao, Y., Zhang, M., & Zhou, S. (2021). Biodegradability of di-(2-ethylhexyl) phthalate by a newly isolated bacterium *Achromobacter* sp. RX. Sci. Tot. Environ., 755, 142476. https://doi.org/10.1016/j.scitotenv.2020.142476

Wang, Z., Xin, X., Shi, X., & Zhang, Y. (2020). A polystyrene-degrading Acinetobacter bacterium isolated from the larvae of *Tribolium* *castaneum*. Sci. Tot. Environ., 726, 138564. https://doi.org/10.1016/j.scitotenv.2020.138564

Zang, H., Dai, Y., Sun, Y., Jia, T., Song, Q., Li, X., Jiang, X., Sui, D., Han, Z., Li, D., & Hou, N. (2020). Mechanism of the biodemulsifier-enhanced biodegradation of phenanthrene by *Achromobacter* sp. LH-1. Colloids and Surfaces. B, Biointerfaces, 195, 111253. https://doi.org/10.1016/j.colsurfb.2020.111253

Zhang, B., Xu, W., Ma, Y., Gao, X., Ming, H., & Jia, J. (2023). Effects of bioaugmentation by isolated *Achromobacter xylosoxidans* BP1 on PAHs degradation and microbial community in contaminated soil. J.Environ. Manag., 334, 117491. https://doi.org/10.1016/j.jenvman.2023.117491
